# GCN2 in the Brain Programs PPARγ2 and Triglyceride Storage in the Liver during Perinatal Development in Response to Maternal Dietary Fat

**DOI:** 10.1371/journal.pone.0075917

**Published:** 2013-10-10

**Authors:** Xu Xu, Jingjie Hu, Barbara C. McGrath, Douglas R. Cavener

**Affiliations:** Department of Biology, Center for Cellular Dynamics and the Huck Institute of the Life Sciences, Penn State University, University Park, Pennsylvania, United States of America; Clermont Université, France

## Abstract

The liver plays a central role in regulating lipid metabolism and facilitates efficient lipid utilization and storage. We discovered that a modest increase in maternal dietary fat in mice programs triglyceride storage in the liver of their developing offspring. The activation of this programming is not apparent, however, until several months later at the adult stage. We found that the perinatal programming of adult hepatic triglyceride storage was controlled by the eIF2α kinase GCN2 (EIF2AK4) in the brain of the offspring, which stimulates epigenetic modification of the *Pparγ2* gene in the neonatal liver. Genetic ablation of *Gcn2* in the offspring exhibited reduced hepatic triglyceride storage and repressed expression of the peroxisome proliferator-activated receptor gamma 2 (*Pparγ2*) and two lipid droplet protein genes, *Fsp27* and *Cidea*. Brain-specific, but not liver-specific, *Gcn2 KO* mice exhibit these same defects demonstrating that GCN2 in the developing brain programs hepatic triglyceride storage. GCN2 and nutrition-dependent programming of *Pparγ2* is correlated with trimethylation of lysine 4 of histone 3 (H3K4me3) in the *Pparγ2* promoter region during neonatal development. In addition to regulating hepatic triglyceride in response to modest changes in dietary fat, *Gcn2* deficiency profoundly impacts the severity of the obese-diabetic phenotype of the leptin receptor mutant (db/db) mouse, by reducing hepatic steatosis and obesity but exacerbating the diabetic phenotype. We suggest that GCN2-dependent perinatal programming of hepatic triglyceride storage is an adaptation to couple early nutrition to anticipated needs for hepatic triglyceride storage in adults. However, increasing the hepatic triglyceride set point during perinatal development may predispose individuals to hepatosteatosis, while reducing circulating fatty acid levels that promote insulin resistance.

## Introduction

The liver plays a central role in lipid homeostasis by temporarily storing excess triglycerides and releasing them into circulation for use in peripheral tissues or for long-term storage in adipose tissue. Excessive accumulation of triglycerides in the liver can result in non-alcoholic fatty liver disease (NAFLD) or the more severe condition of non-alcoholic steatohepatitis (NASH). Although fatty liver diseases are frequently associated with insulin resistance [1], a deficiency in the ability to store triglycerides in the liver can also lead to increased lipid concentrations elsewhere resulting insulin resistance [2], [3]. Thus regulating hepatic triglyceride storage in concert with whole body metabolism requires a dynamic balance. Triglycerides are stored in lipid droplet organelles in the liver in a similar fashion as previously described for adipose tissue [4]–[6]. Several lipid droplet proteins including the PAT family (perilipin 1, perilipin 2/ADRP, and perilipin 3/TIP47) and CIDE family (CIDEA, CIDEB, and CIDEC/FSP27) were first identified in adipose tissue and shown to orchestrate storage and retrieval of triglycerides [7], [8] and mutations in some of these genes result in lipodystrophy and insulin resistance [9].

FSP27 and CIDEA, which act together to promote unilocular lipid droplets in adipose tissue [4], [6], [10]–[13], were previously shown to be expressed at low levels in the liver of individuals consuming a low fat diet, but are strongly induced in the liver of leptin deficient mice, which develop fatty liver [14]. Hepatosteatosis associated with obese *ob/ob* mice is also improved when FSP27 expression is reduced in the liver whereas overexpression of FSP27 in hepatocytes increases triglyceride accumulation [14], [15]. PPARγ2, which is essential for differentiation of adipocytes, is a key regulator of the FSP27 and CIDEA genes in adipose tissue and the liver [14], [16], [17]. The *Pparγ2* promoter is particularly sensitive to nutritional changes [18]–[20] and is induced in the liver of the leptin deficient obese mouse. More recently it was reported that ablating *PPARγ* exclusively in the liver blocks the development of hepatosteatosis and induction of FSP27 in the liver of leptin deficient (*ob/ob*) mice [2], [14]. However these mice exhibit hypertriglyceridemia and more severe hyperglycemia, suggesting that storage of triglycerides in the liver during lipid overload is normally protective.

Dyslipidemia is impacted by diet, but also may be influenced by maternal nutrition and obesity during perinatal development [21]–[29]. The effect of nutrition on maternal programming can be imprinted in the developing brain of the offspring through epigenetic modifications [30]–[33], however the identity of the regulatory factors in the brain that control maternal programming of peripheral tissues have yet to be discovered.

GCN2 eIF2 alpha kinase (EIF2AK4) was first discovered as a key sensor of amino acid deprivation that acts in yeast to upregulate amino acid biosynthesis [34]–[36]. Mice deficient for GCN2 exhibit increased fetal mortality if the maternal diet is lacking an essential amino acid [37]. GCN2 also plays a key role in the brain to sense amino acid levels and regulate feeding behavior [38], [39]. More recently GCN2 was implicated in regulating lipid metabolism in mice fed a diet deficient in leucine [40]. The role of GCN2 in regulating fat metabolism, however, has not been investigated beyond its important function in adapting to amino acid deprivation. We report herein that GCN2 in the developing brain is responsible for programming *Pparγ2*, *Fsp27*, and *Cidea* gene expression and triglyceride storage in the liver as a function of perinatal nutrition.

## Materials and Methods

### Animals, Diets and Experiments

Wild type *C57BL/6J* mice were obtained from the Jackson Laboratory. *Gcn2 KO* and *Gcn2 floxed* mice were generated as previously described [37] and subsequently backcrossed eight or more generations into a *C57BL/6J* background. *Liver-specific Gcn2 KO* (*LiGcn2 KO*) and *brain-specific Gcn2 KO* (*BrGcn2 KO*) mice were generated by crossing the Albumin-Cre and Nestin-Cre transgenic mice to the *Gcn2* floxed mouse. *C57BL/6J-Lepr^db^* heterozygous mice (*db/+*) were purchased from Jackson Laboratory and crossed with *Gcn2 KO* mice to generate *Gcn2;db DKO* (*Gcn2−/−; db/db*) mice.

All mouse strains were maintained on a 12 hr light/dark cycle and were provided free access to 22% (kcal %) fat rodent chow (5020, LabDiet) and tap water prior to the experiments, unless otherwise indicated. The mice used for experiments were 6–8 months of age unless otherwise indicated. The 13% (kcal %) fat rodent chow (5001, LabDiet) was also used for some experiments. For the low fat versus very high fat diet feeding experiment, rodent diet with 10% (kcal %) fat and 60% (kcal %) fat were purchased from Research Diets, Inc. For rosiglitazone feeding experiment, rosiglitazone (Sigma) was mixed with transgenic dough diet, bacon flavor (BioServ) and made into ∼125 mg small pills. Mice were fed with rosiglitazone containing pills with a dosage of ∼3 mg/kg/day for two weeks. All animal experiments were approved by the Pennsylvania State University Institutional Animal Care and Use Committee.

### Serum and Liver Metabolites Measurements

Mice were sacrificed by CO_2_ euthanization. Serum was obtained by centrifugation of blood samples at 10,000 rpm/5 min. Tissues were resected and snap frozen in liquid nitrogen and stored at −80°C for further analysis. Serum glycerol, triglyceride, total cholesterol, and free fatty-acid levels were determined enzymatically using glycerol reagent (Sigma), triglyceride reagent (Sigma), cholesterol/cholesteryl ester quantification kit (BioVision), and Free Fatty acids, half micro test kit (Roche), respectively. Serum insulin and leptin were measured using the mouse insulin/leptin assay kit (MSD). Serum HDL and LDL/VLDL were separated by precipitation buffer (HDL and LDL/VLDL Cholesterol Quantification Kit, BioVision) and then triglyceride and cholesterol contents were quantified as mentioned above. Liver lipids were extracted from ∼80 mg liver samples as previously described [41] for triglyceride assay. For cholesterol assay, liver lipids were extracted with chloroform: isopropanol: NP-40 (7∶11∶0.1) and dissolved in 200 μl cholesterol assay buffer according to instructions of the assay kit. Values were calculated as mg/g of wet tissue.

### Liver Fatty Acid Oxidation Assay

Peroxisome-mediated fatty acid β-oxidation activity was measured as previously described [42], [43]. Briefly, 50 mg livers were homogenized in 500 μl cold 0.25 M sucrose, centrifuged at 600× g for 10 min and the upper lipid layer was aspirated. 50 μl of 10% Triton X-100 was added to each 450 μl sample supernatant. The samples were then assayed for peroxisomal β-oxidation with or without potassium cyanide, which inhibits mitochondrial β-oxidation. The oxidation of palmitoyl-CoA was quantified spectrophotometrically by measuring the reduction of nicotinamide adenine dinucleotide-positive at 340 nm. The rate of NAD+ reduction is equivalent to the rate of acetyl-CoA formed, which reflects fatty acid oxidation activity.

### Lipolysis Measurement

Adipocytes were isolated from abdominal fat pads according to the classical Rodbell method with modifications, such as the addition of adenosine in digestion solution and wash buffer to suppress lipolysis during isolation [44]. Briefly, abdominal fat tissues weighing about 800 mg were dissected from mice immediately after sacrificing and put into a scintillation vial with 200 ml digestion solution. Fat pads were minced immediately to pieces in about 1mm diameter and incubated in a shaking water bath at 37°C for 30–40 min at the speed of 220 rpm. The digested mixture was filtered through gauze into a 50 ml conical polypropylene tube and allowed to stand for 5 min. The infranatant containing the digestion solution was aspired with a long needle and the remaining floating layer of adipocytes was washed three times with 10 ml adenosine-replete adipocyte washing buffer. To determine lipolysis activity, the isolated adipocytes were resuspended with 1 ml adipose incubation buffer and incubated in a water bath at 37°C for 1 hour with gentle shaking. After incubation, 200 μl of infranatant was taken and assayed for glycerol release using the glycerol reagent (Sigma). Lipolysis activity is the amount of glycerol (mg) released per gram of adipose tissue per hour.

### Lipoprotein Lipase Assay

Lipoprotein lipases were extracted from tissues as previously described [45]. Briefly, 50 mg frozen tissue was homogenized in 500 μl Krebs-Ringer solution, 0.1 M Tris-HCl buffer (PH 8.4) containing 1 g/100 ml of BSA and 2.5 U (50 mg/l) of heparin and incubated at 28°C for 40 min with gentle shaking. Tissue remains were removed by centrifugation for 5 min and the supernatant was used for the measurement of lipoprotein lipase activity with the Roar LPL Activity Assay kit, which includes a non-fluorescent substrate emulsion that becomes intensely fluorescent upon interaction with LPL. The lipoprotein lipase activities were calculated as the amount of hydrolyzed substrates (μmol) generated per ml of tissue homogenate.

### Oil Red O, Hematoxylin & Eosin and Immunofluorescence Staining

Liver pieces were fixed in 10% formalin solution for 2 hours at room temperature, transferred to 20% sucrose at 4°C overnight, and then were embedded in Shandon Cryomatrix (Thermo) for cryosectioning. Frozen sections of liver (5 μm) were stained with oil red O for lipids or anti-CIDEA antibody (Santa Cruz) for IF labeling of CIDEA proteins. Formalin-fixed, paraffin-embedded sections (5 μm) of liver were stained with hematoxylin and eosin for histological analysis.

### RNA Isolation and Relative Quantitative RT-PCR

RNA was extracted with Qiagen RNeasy® Mini Kit (Qiagen) from tissues or cell cultures. RNA was quantitated by Quant-It TM RiboGreen® RNA Assay Kit (Invitrogen). 1 µg RNA was used for reverse transcription with qScriptTM cDNA supermix (Quanta) to generate cDNA in a 20 μl reaction volume. Quantitative RT-PCR was performed with qPCR core kit for SYBR® Green I (Eurogentec) by 7000 Sequence detection system (Applied Biosystems). GAPDH was coamplified with genes of interest as an internal control. The cycle differences with GAPDH are used to determine the relative intensity of genes of interests. Primers used for real-time PCR are listed in Table S2.

### Western Blot Analysis

Whole cell lysates were extracted with RIPA buffer containing protease inhibitor cocktail (Sigma), phosphatase inhibitor cocktail 1, 2 (Sigma). Primary antibodies for PPARα, PPARγ, C/EBPβ (Santa Cruz), FSP27 (Abcam), and α-tubulin (Sigma) were used.

### Chromatin Immunoprecipitation

ChIP was performed according to the Farnham Laboratory ChIP protocol (Dr. Peggy Farnham, UC Davis, personal communication) for tissues with modifications. Briefly, the cross-linked chromatin was extracted from frozen liver samples and sheared by sonication to 500–1000 bp fragments and ChIP was performed with ChIP assay kit (Millipore). Immunoprecipitation was performed with ∼3 µg anti-H3K4me3, H3K9me3 and H3K27me3 (Abcam) antibodies and rabbit IgG (sigma). Precipitated genomic DNA was quantitated by realtime PCR with primers for *Pparγ1* or *Pparγ2* promoter regions, as previously described [46]. Data was expressed as percentage recovery of input DNA.

### Data Analysis

All data are expressed as mean ± SEM for experiments including numbers of mice as indicated. The two-tailed Student's test was used to evaluate statistical differences between wild type mice and *Gcn2 KO* mice in random fed or fasted state unless being specially indicated.

## Results

### GCN2 deficiency alters hepatic and circulating lipid homeostasis

**Figure 1:**
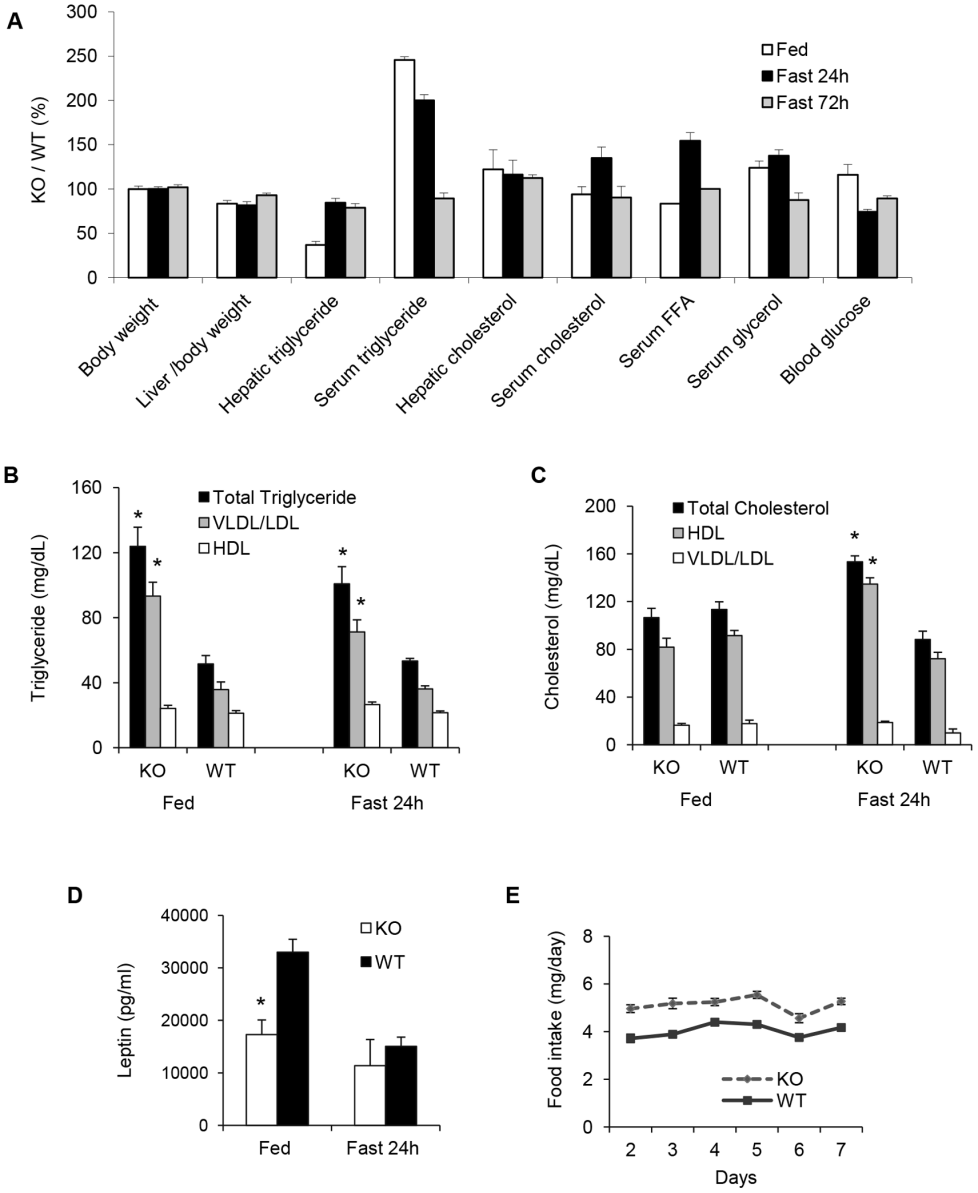
*Gcn2* deficient mice display reduced hepatic triglyceride but increased serum triglyceride levels. (A). A comparison of phenotypic and metabolic parameters between wild type (WT) and *Gcn2 KO* (KO) mice, 8 months of age, in random fed state and in response to starvation (Data expressed as a percentage of KO mice to WT mice (see Table S1 for parameter values). (B). Triglyceride content in the VLDL/LDL and HDL fractions of the serum of wild type (WT) and *Gcn2 KO* (KO) mice (mean ± SEM, n = 8, *p<0.05 *Gcn2 KO vs.* WT). (C). Cholesterol content in the VLDL/LDL and HDL fractions of the serum in mice of the indicated genotypes (mean ± SEM, n = 8, *p<0.05 *Gcn2 KO vs.* WT). (D). Serum leptin concentration of mice of indicated genotypes (mean ± SEM, n≥8, *p<0.05 *Gcn2 KO vs.* WT). (E). Daily food intake of mice of the indicated genotypes fed ad libitum for one week (mean ± SEM, n≥8).

Metabolic and morphometric parameters were measured in 8-month old *Gcn2 KO* and WT mice reared on a standard breeding chow diet (5020, Lab Diet) which is composed of 22 kcal% fat denoted here as medium fat chow (MFC). Body weight and adiposity of *Gcn2 KO* mice were normal, but they exhibited reduced liver weight, a 3-fold reduction in hepatic triglycerides (Fig. 1A and Table S1), and 2.5 times the normal level of serum triglycerides in the random fed state. Increased VLDL was correlated with the hypertriglyceridemia in *Gcn2 KO* mice (Fig. 1B). Serum cholesterol and HDL levels were normal in random fed *Gcn2 KO* mice but were elevated in fasted mice in contrast to the expected modest decline in wildtype mice (Fig. 1C). In addition *Gcn2 KO* mice exhibited decreased serum leptin (Fig. 1D) and increased food intake (Fig. 1E) in the random fed state, without impacting body weight or adiposity. After being subjected to a 24 h fasting period, *Gcn2 KO* mice showed no significant differences in body and adipose tissue weight however the differences in liver size between genotypes persisted (Fig. 1A and Table S1). The 3-fold reduction in hepatic triglyceride content seen in random fed *Gcn2 KO* mice was largely erased after a 24-fast and the 2.5-fold elevation of serum triglycerides was eliminated after a 72 hour fast.

### Hypertriglyceridemia and reduced hepatic triglycerides in *Gcn2* deficient mice are due to dysfunctions in lipid handling controlled by PPARγ and lipid droplet proteins

The hypertriglyceridemia and reduced liver triglyceride (HiS/LoH TG) phenotype of the *Gcn2 KO* mice in the fed state suggested an imbalance in lipid handling. Several possible causes of hypertriglyceridemia in *Gcn2 KO* mice were examined including defects in lipid export or import from the circulatory system. Lipolysis of primary adipocytes was measured and found to be normal (Fig. S1A). The activities of key lipoprotein lipases responsible for lipid uptake into peripheral tissues were normal in the liver, adipose tissue, and muscle of *Gcn2 KO* mice (Fig. S1B–D). Expression of genes that regulated lipid uptake and secretion in the liver was also found not to be significantly different in *Gcn2* deficient mice (Fig. S1E–F).

The cause of the marked reduction in stored triglycerides in the liver was explored by examining the metabolism of lipids within the liver. Increased oxidation of fatty acids in the liver could possibly result in a reduced level of stored triglycerides, however *Gcn2 KO* mice displayed significantly reduced rates of fatty acid oxidation in the random fed state (Fig. S2A). When subjected to 24 fasting, fatty acid oxidation rates were elevated in both genotypes as expected, and the relative differences between genotypes was reversed. Consistent with reduced beta-oxidation in the fed state, the expression of the microsomal (*Cyp4A14, Cyp4A10, Cyp2b10, Cyp4A12a,* and *Aldh3a2*) and peroxisomal fatty acid oxidation (*Ehhadh* and *Acaa1a*) related genes was reduced in livers of *Gcn2 KO* mice (Fig. S2B). However, PPARα and most of the key mitochondrial fatty acid oxidation genes did not show significant genotypic differences (Fig. S2C–D). Hepatic lipogenic gene expression was unaltered in *Gcn2 KO* mice (Fig. S2E) suggesting that decreased lipid synthesis was not the cause of reduced hepatic triglycerides although we did not directly measure lipid synthesis. *Gcn2* deficient mice also exhibited normal suppression of lipogenic gene expression in response to fasting (Fig. S2E).

**Figure 2:**
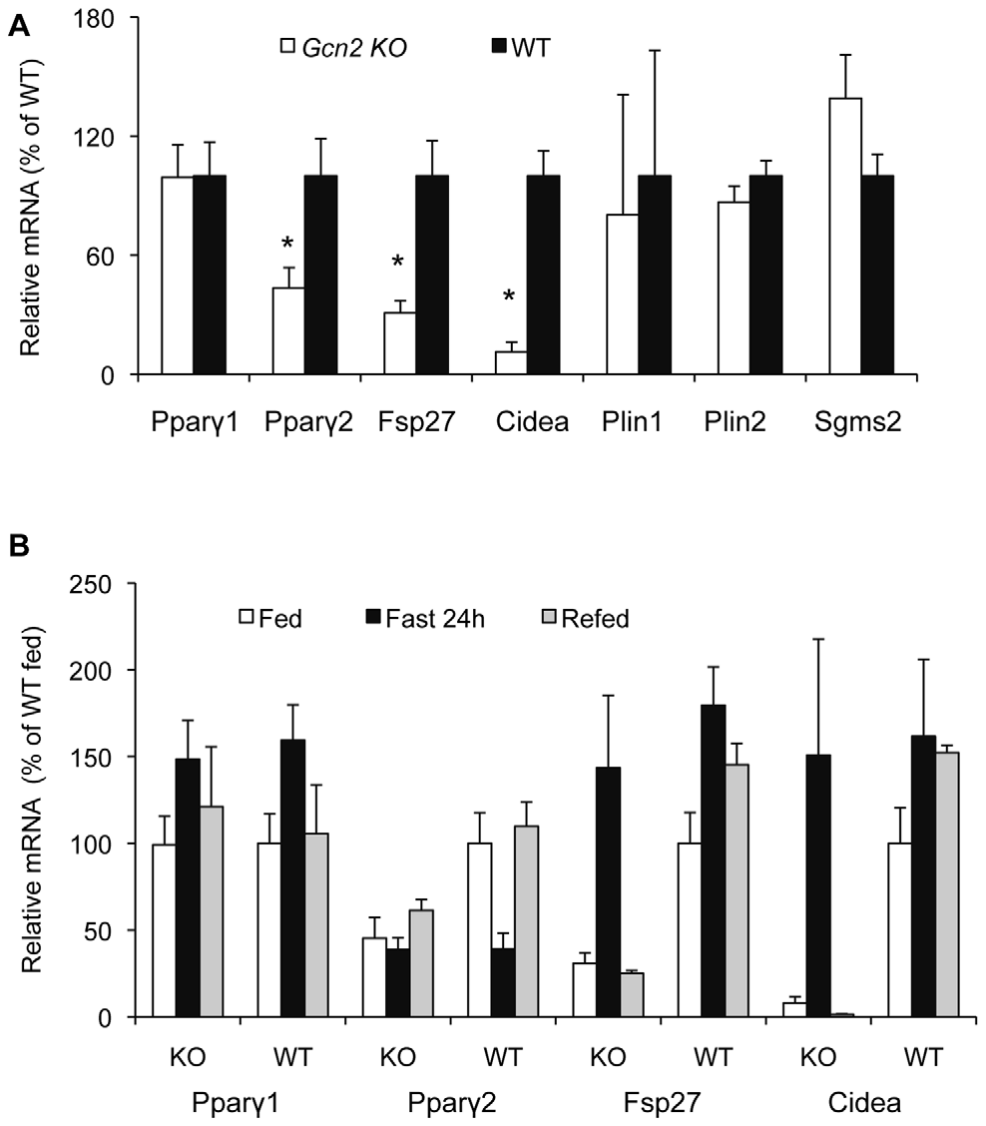
GCN2 regulates basal expression of PPARγ2 and lipid droplet proteins FSP27 and CIDEA in the liver but not the fasting response. (A). Expression of *Pparγ1*, *Pparγ2, Fsp27*, *Cidea*, *Plin1, Plin2 and Sgms2* mRNAs in livers of wild type (WT) and *Gcn2 KO* (KO) mice, 8 months of age, (normalized to WT mice, mean ± SEM, n = 8, *p<0.05 *Gcn2 KO vs*. WT). (B). Expression of *Pparγ1*, *Pparγ2, Fsp27* and *Cidea* mRNAs in livers of random fed, 24 hr-fasted and refed wild type (WT) and *Gcn2 KO* (KO) mice (normalized to WT mice, mean ± SEM, n = 8, *p<0.05 *Gcn2 KO vs*. WT).

In the absence of evidence that dysfunctions in lipid metabolism could explain the HiS/LoH TG phenotype, we speculated that misregulation of hepatic triglyceride storage could underlie both the decreased hepatic triglyceride content and hypertriglyceridemia. A similar phenotype has been described for liver-specific *Pparγ KO* mice with a leptin deficiency [2], [14] and hence we examined the expression of *Pparγ* and the factors that promote triglyceride storage within lipid droplets (LD). The expression of *Pparγ2, Fsp27,* and *Cid*ea mRNA was reduced by 40%, 75%, and 85%, respectively in the liver of *Gcn2 KO* mice (Fig. 2A) whereas the expression of *Pparγ1, Plin1, Plin2*, and *Sgms2* was normal. In contrast, *Pparγ1,2* and these LD protein genes were not significantly reduced in the adipose tissue of the abdominal fat pads (Fig. S3A). The large differences in the expression of *Pparγ2, Fsp27*, and *Cidea* in the liver of *Gcn2 KO* mice were erased when animals were fasted for 24 hours (Fig. 2B) consistent with the reversal of the HiS/LoH TG phenotype (Fig. 1A). Upon refeeding, however, the HiS/LoH TG phenotype and repression of *Pparγ2, Fsp27*, and *Cidea* mRNAs in the liver was restored in *Gcn2* KO mice (Fig 2B). Although the fasting induction of Fsp27 and Cidea is independent of GCN2 and PPARγ2, these data suggest that GCN2 and PPARγ2 programs their expression relative to the fed state and this program is re-established following a fasting episode.

### GCN2 regulates an adaptive response to dietary fat to increase hepatic triglyceride storage

**Figure 3:**
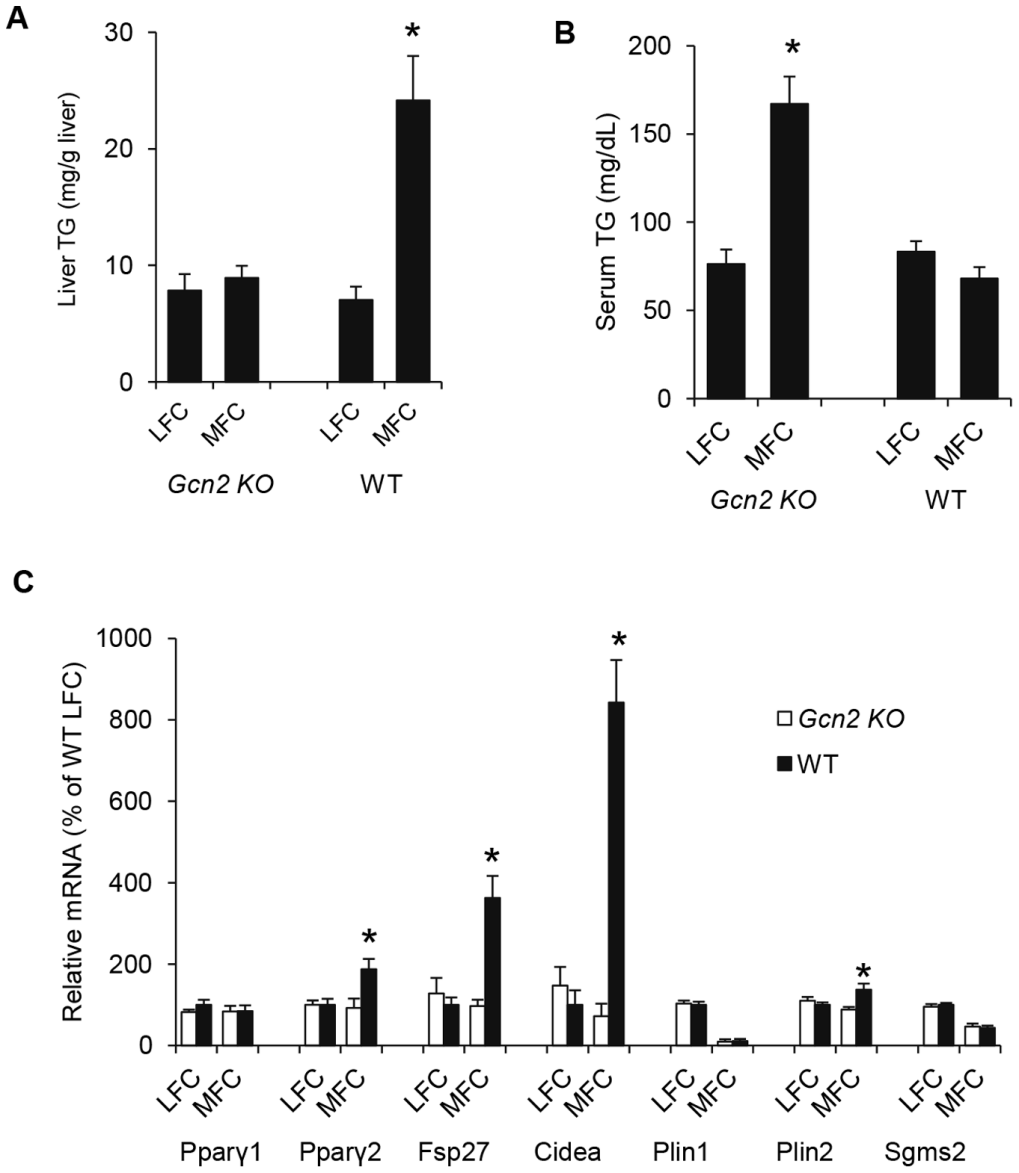
GCN2 regulation of liver and serum triglycerides and expression of PPARγ2 and lipid droplet proteins FSP27 and CIDEA in the liver is dependent upon dietary fat. (A). Liver triglyceride (TG) content of mice of indicated genotypes after Low Fat Chow (LFC) and Medium Fat Chow (MFC) feeding (mean ± SEM, n = 8, *p<0.05 *Gcn2 KO vs*. WT). (B). Serum triglyceride (TG) level of mice of indicated genotypes after LFC and MFC feeding (mean ± SEM, n = 8, *p<0.05 *Gcn2 KO vs*. WT). (C). Expression of *Pparγ1*, *Pparγ2, Fsp27*, *Cidea*, *Plin1, Plin2 and Sgms2* mRNAs in livers of mice of indicated genotypes fed with LFC and MFC (normalized to wildtype mice on LFC, mean ± SEM, n = 4, *p<0.05 *Gcn2 KO vs*. WT).

FSP27 and CIDEA are highly expressed in adipocytes where they are important in regulating the formation of unilocular lipid droplets from multilocular lipid droplets [6], [11]. In contrast these two genes are expressed at very low levels (ca. 25-fold less) in the liver, but have been reported to be induced under high lipid load associated with a block in leptin signaling [5]. We suspected that the medium fat chow (MFC) diet containing 22% Kcal fat, which we had used throughout this study, contained sufficiently elevated levels of triglycerides to induce the expression of *Fsp27* and *Cidea* in wild type mice beyond the level seen in mice reared on standard low fat chow (LFC) that contains 13% Kcal fat. Wild type mice breed and reared on MFC exhibited substantially higher TG levels and higher expression of *Pparγ2*, *Fsp27*, *Cidea*, compared to mice breed and reared on LFC (Fig. 3A,C). In contrast, *Pparγ*2, *Fsp27* and *Cidea* mRNA expression and hepatic triglyceride content was not induced in *Gcn2 KO* mice reared on the MFC diet; however these mice did exhibit abnormally high serum TG. The level of *Pparγ*2, *Fsp27* and *Cidea* gene expression and the level of serum and hepatic TG in LFC-reared wild type and *Gcn2 KO* mice is similar (Fig. 3A–C) demonstrating that the HiS/LoH TG phenotype is dependent upon increased fat present in the MFC diet. These findings suggest that GCN2 senses elevated dietary fat levels and acts to increase hepatic triglyceride storage.

**Figure 4:**
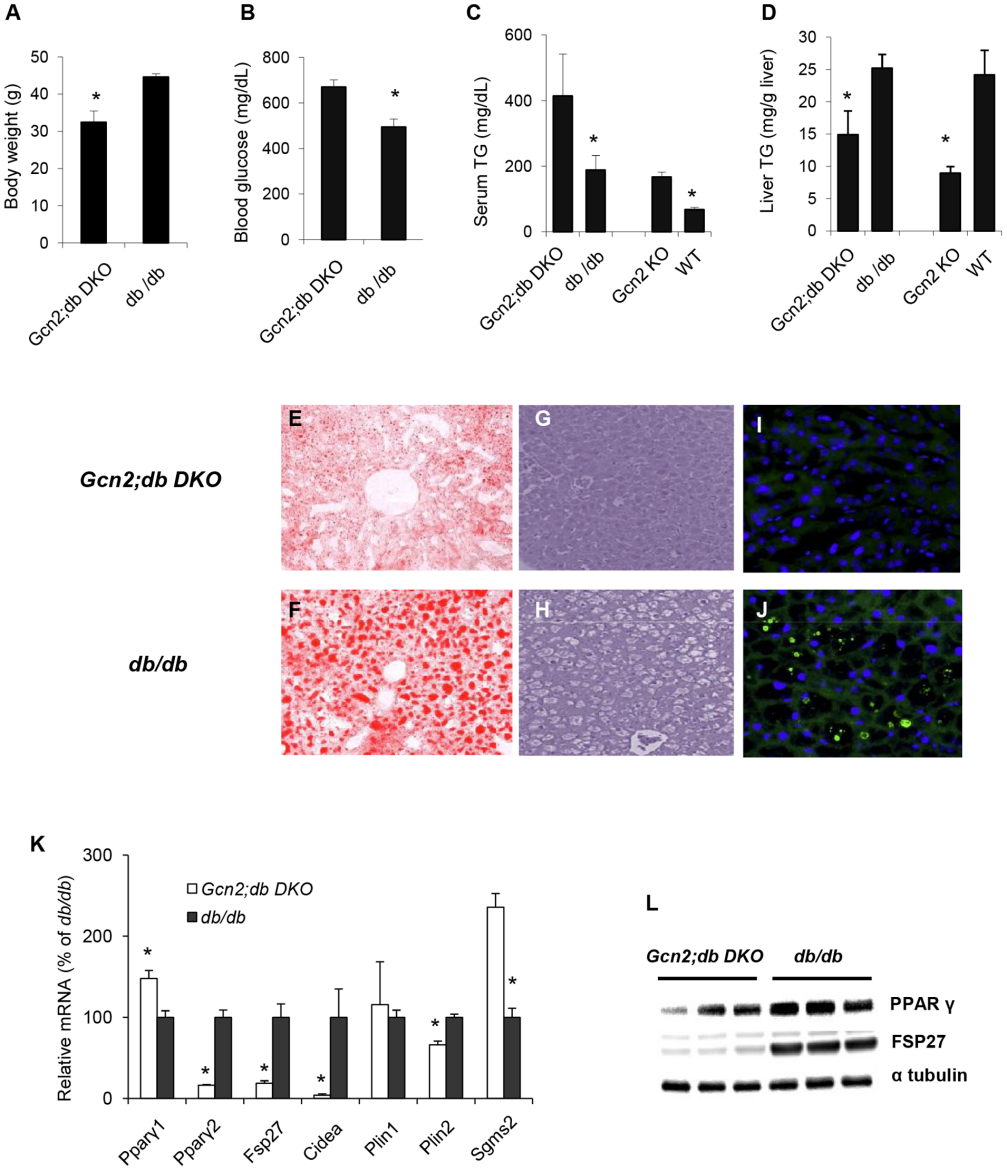
A block in leptin signaling amplifies the HiS/LoH triglyceride phenotype of Gcn2 mice and hyperglycemia of the db/db mice. (A). Body weight (B) Blood glucose (C) Serum triglyceride (D) Liver triglyceride of *Gcn2;db DKO* (*Gcn2 −/−; db/db*) and *db/db* (*Gcn2+/+;db/db*) mice (mean ± SEM, n = 4, *p<0.05, *Gcn2;db DKO* versus *db/d*b mice). (E). Oil red O staining of representative liver sections of *Gcn2;db DKO* mice and (F) *db/db* mice. (G). H&E staining of representative liver sections of *Gcn2;db DKO* mice and (H) *db/db* mice. Unstained empty areas indicated formation of lipid droplets. (I). mmunofluorescent (IF) staining of CIEDA proteins (green) in representative liver sections of *Gcn2;db DKO* mice and (J) *db/db* mice. Nuclei are stained with DAPI (blue). (K). Expression of *Pparγ1*, *Pparγ2, Fsp27*, *Cidea*, *Plin1, Plin2 and Sgms2* mRNAs in livers of mice of indicated genotypes (normalized to *db/db* mice, mean ± SEM, n = 8, *p<0.05, *Gcn2;db DKO* vs. *db/db*). (L). PPARγ and FSP27 protein western blotting from whole liver lysates of mice of indicated genotypes.

To further explore the role of GCN2 in regulating lipid handling under conditions of excess lipid load, the leptin receptor mutation (*db*) was introduced into the background of *Gcn2* deficient mice to generate *Gcn2*;*db* double knockout (DKO) mice. *Gcn2;db DKO* mice exhibited 40% reduction in body weight (Fig. 4A) compared to *db/db* mice, which showed the expected extreme obese phenotype. Although less obese, the *Gcn2;db DKO* mice progressed much more rapidly to hyperglycemia (Fig. 4B). Of particular relevance, serum triglyceride levels were doubled in *Gcn2;db DKO* mice (Fig. 4C) compared to the very high levels seen in *db/db* mice whereas the elevated hepatic triglycerides (Fig. 4D) and extreme liver steatosis typically seen in *db/db* mice was substantially ameliorated (Fig. 4E–G compared to Fig. 4F-H). Moreover, CIDEA containing lipid droplets were readily seen in *db/db* mice but undetectable in *db/db; Gcn2 KO* mice (Fig. 4I,J). *Pparγ2, Fsp27*, and *Cid*ea mRNAs were dramatically repressed in the liver of *Gcn2;db DKO* mice compared to *db/db* mice (Fig. 4K). PPARγ and FSP27 protein levels in *Gcn2;db DKO* mice exhibited a substantial reduction with FSP27 being almost entirely eliminated (Fig. 4L). In contrast to the liver, the expression of *Pparγ2, Fsp27*, and *Cidea* in WAT was not repressed in *Gcn2;db DKO* mice (Fig. S3B).

### Maternal diet programs hepatic triglyceride storage in offspring

**Figure 5:**
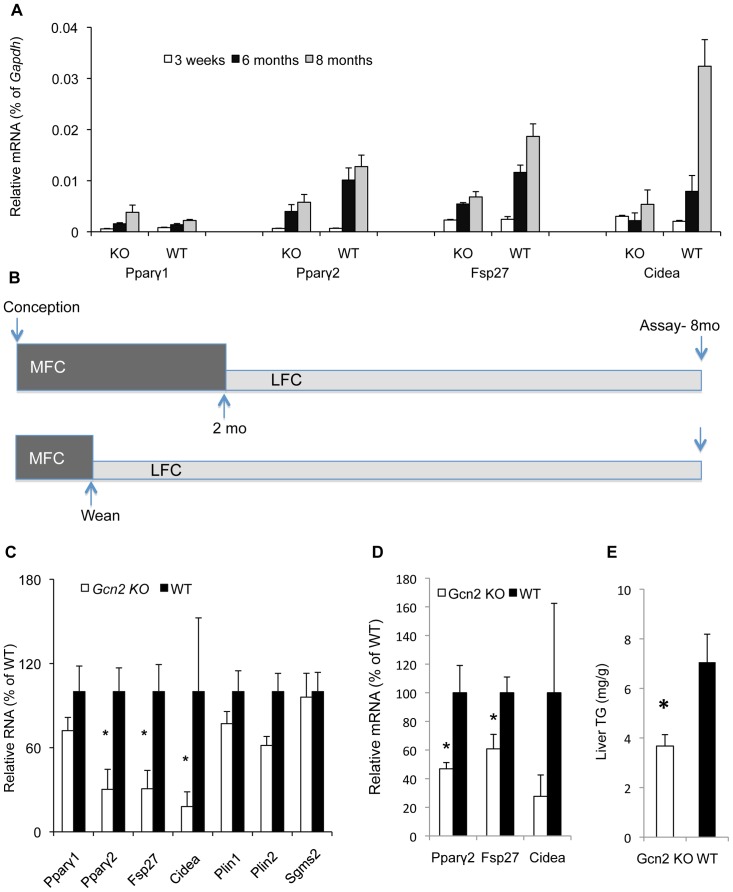
The nutritional programming of PPARγ2, FSP27, and CIDEA by GCN2 is fixed during perinatal development and resistant to nutritional changes at the adult stage. (A). *Pparγ1*, *Pparγ2, Fsp27* and *Cidea* mRNA levels relative to *Gapdh* mRNA in livers of 3 weeks, 6 months and 8 months old wild type (WT) and *Gcn2 KO* (KO) mice (mean ± SEM, n = 4, *p<0.05 *Gcn2 KO vs*. WT). (B). Illustration of time points for diet shift experiments. Upper panel illustrates the diet shift from MFC diet to LFC occurred two months of age. Lower panel illustrates the diet shift from MFC to LFC diet occurred at weaning (3 weeks of age). Mice in both experiments were euthanized at 8 months of age for analysis of serum and hepatic triglycerides and gene expression. (C). Expression of *Pparγ1*, *Pparγ2, Fsp27*, *Cidea*, *Plin1, Plin2 and Sgms2* mRNAs in livers of mice of indicated genotypes, which were reared on MFC chow diet from conception to 2 months of age and then switched to the lower fat chow diet (LFC) for additional 6 months (normalized to WT mice, mean ± SEM, n = 8, *p<0.05 *Gcn2 KO vs*. WT). (D). Expression of *Pparγ2, Fsp27*, *Cidea* mRNAs in livers of mice of indicated genotypes, which were reared on MFC chow diet before weaning and then switched to the lower fat chow diet (LFC) until 8 months old (normalized to WT mice, mean ± SEM, n = 8, *p<0.05 *Gcn2 KO vs*. WT). (E). Liver triglyceride content in mice of indicated genotypes, which were reared on MFC chow diet before weaning and then switched to the lower fat chow diet (LFC) until 8 months old (normalized to WT mice, mean ± SEM, n = 8, *p<0.05 *Gcn2 KO vs*. WT).

The impact of excess dietary lipids on the hepatic triglyceride storage and gene expression was investigated as a function of developmental stage. At weaning *Pparγ2, Fsp27*, and *Cidea* are expressed at very low levels in the liver but increase several fold in mature wild type adults reared on MFC (Fig. 5A). At the age of 6–8 months old, the suppression of these genes in the liver is apparent in *Gcn2 KO* mice and this corresponds to the earliest time point that we have observed substantial genotypic differences in hepatic triglycerides.

Metabolic phenotypes manifested at the adult stage may be strongly impacted by earlier nutritional experience during development [47]. To determine if early dietary exposure to MFC diet has programming effect on adult lipid droplet gene expression and TG levels, mice were reared on MFC chow diet from conception to 2 months of age and then switched to the lower fat chow diet (LFC) for an additional 6 months (Fig. 5B upper panel). *Gcn2* genotype differences similar to mice that were reared and maintained continuously on the MFC diet were seen for *Pparγ2*, *Fsp27*, and *Cidea* gene expression (Fig. 5C). Thus *Gcn2* genotype sets the LD gene expression program during development as a function of dietary fat content several months in advance of when either the gene expression or hepatic triglyceride differences are first apparent.

To further delineate the developmental programming of LD gene expression and triglyceride levels, mice were reared on MFC diet during gestation and neonatal development and then switched to LFC diet at weaning (i.e. 3 weeks old) and then maintained on LFC until 8 months of age (Fig 5B lower panel). Even though these mice were only exposed to the MFC in utero and during neonatal development, *Gcn2* genotype differences were still seen in LD gene expression and hepatic TG levels at 8 month old in contrast to mice exposed only to LFC diet throughout life (Fig. 5D–E). However, serum triglycerides levels were equivalent in wildtype and *Gcn2* KO mice exposed early to MFC diet (not shown), suggesting that this parameter may require a longer programming period. In addition the gene expression differences between genotypes in mice exposed to MFC until weaning versus until 2 months old were not as large, indicating that maximal MFC diet hepatic programming may extend until two–months of age.

### PPARγ2 mediates GCN2-dependent perinatal programming of hepatic triglyceride storage

**Figure 6:**
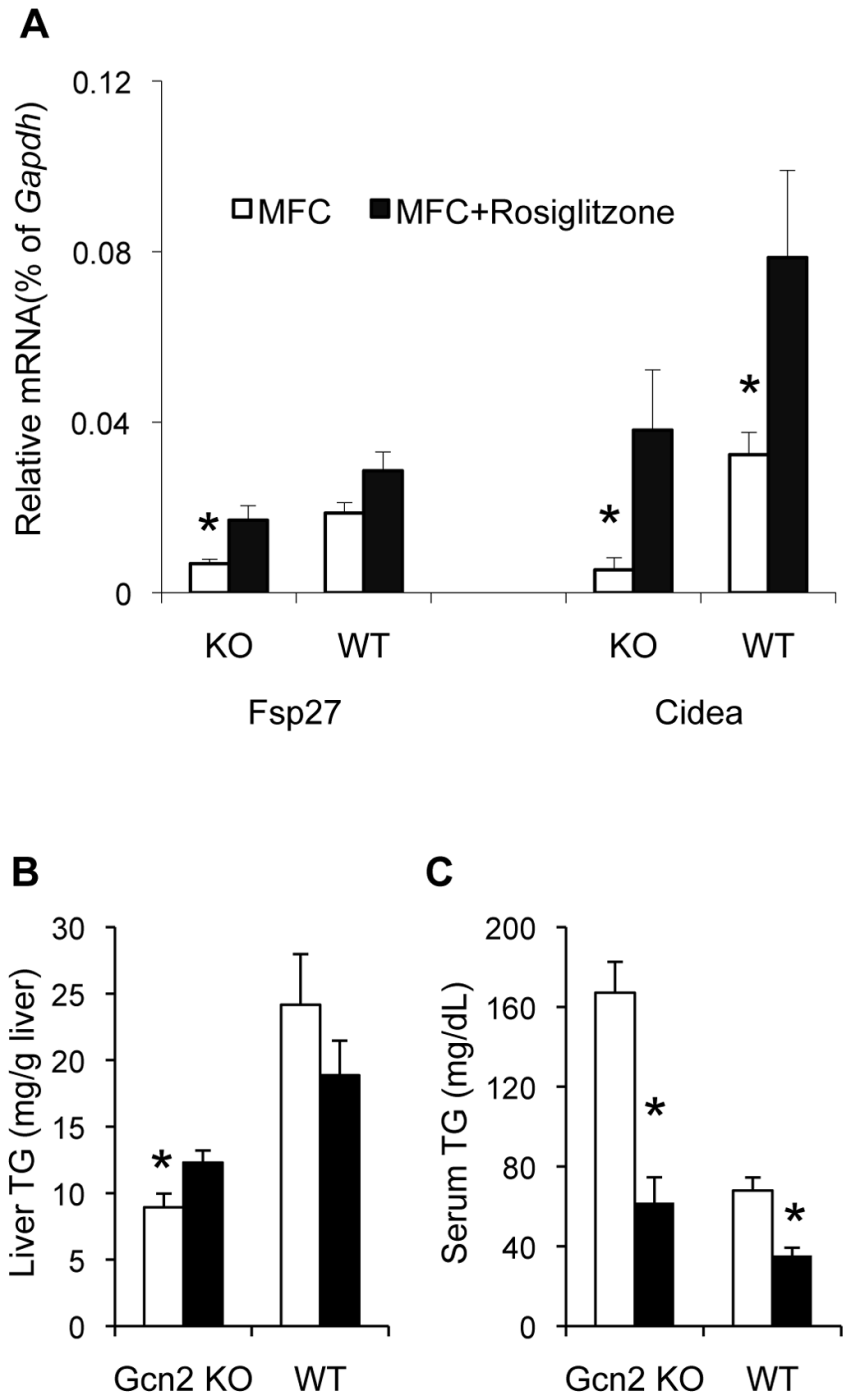
The PPARγ agonist rosiglitazone partially reverses the effect of GCN2 deficiency on liver and serum triglycerides and lipid droplet protein expression. (A). *Fsp27* and *Cidea* mRNA levels in livers relative to *Gapdh* mRNA of *Gcn2 KO* (KO) and wild type (WT) mice after rosiglitazone feeding rosiglitazone or vehicle treated Medium Fat chow diet (MFC) for 3 weeks (mean ± SEM, n = 3–7, *p<0.05 *Fsp27* and *Cidea Gcn2 KO* rosiglitazone *vs*. MFC, *p<0.05 *Cidea Gcn2 WT* rosiglitazone *vs*. MFC, *p<0.05 *Fsp27* and *Cidea* rosiglitazone treated *Gcn2 KO vs*. WT). (B). Liver triglyceride of *Gcn2 KO* (KO) and wild type (WT) mice after rosiglitazone feeding 3 weeks (mean ± SEM, n = 3–7, *p = 0.08 *Gcn2 KO* rosiglitazone *vs*. MFC, *p<0.05 rosiglitazone treated *Gcn2 KO vs*. WT).(C). Serum triglyceride of *Gcn2 KO* (KO) and wild type (WT) mice after rosiglitazone feeding 3 weeks (mean ± SEM, n = 3–7, *p<0.05 *Gcn2 KO* rosiglitazone *vs*. MFC, *p<0.05 *Gcn2 WT* rosiglitazone *vs*. MFC, *p<0.05 rosiglitazone treated *Gcn2 KO vs*. WT).

We reasoned that if PPARγ2 was mediating the GCN2-dependent perinatal programming, we could reverse the HiS/LoH TG phenotype and repression of *Fsp27* and *Cidea* by treating *Gcn2 KO* mice with the PPARγ agonist rosiglitazone and thereby compensate for its reduced level. *Gcn2 KO* mice reared on MFC for eight months and then treated with rosiglitazone for 2 weeks exhibited partial reversal of the HiS/LoH TG phenotype and *Fsp27* and *Cide*a mRNA expression (Fig. 6A–C). Rosiglitazone treatment affected a 2.5 and 7.1 fold increase in *Fsp27* and *Cidea* mRNA expression, respectively, in *Gcn2 KO* mice, which elevated the expression of these genes to a level equivalent to wild type mice reared on a MFC diet. Serum TG levels were more strongly impacted by rosiglitazone treatment than hepatic TG levels (Fig. 6B–C).

**Figure 7:**
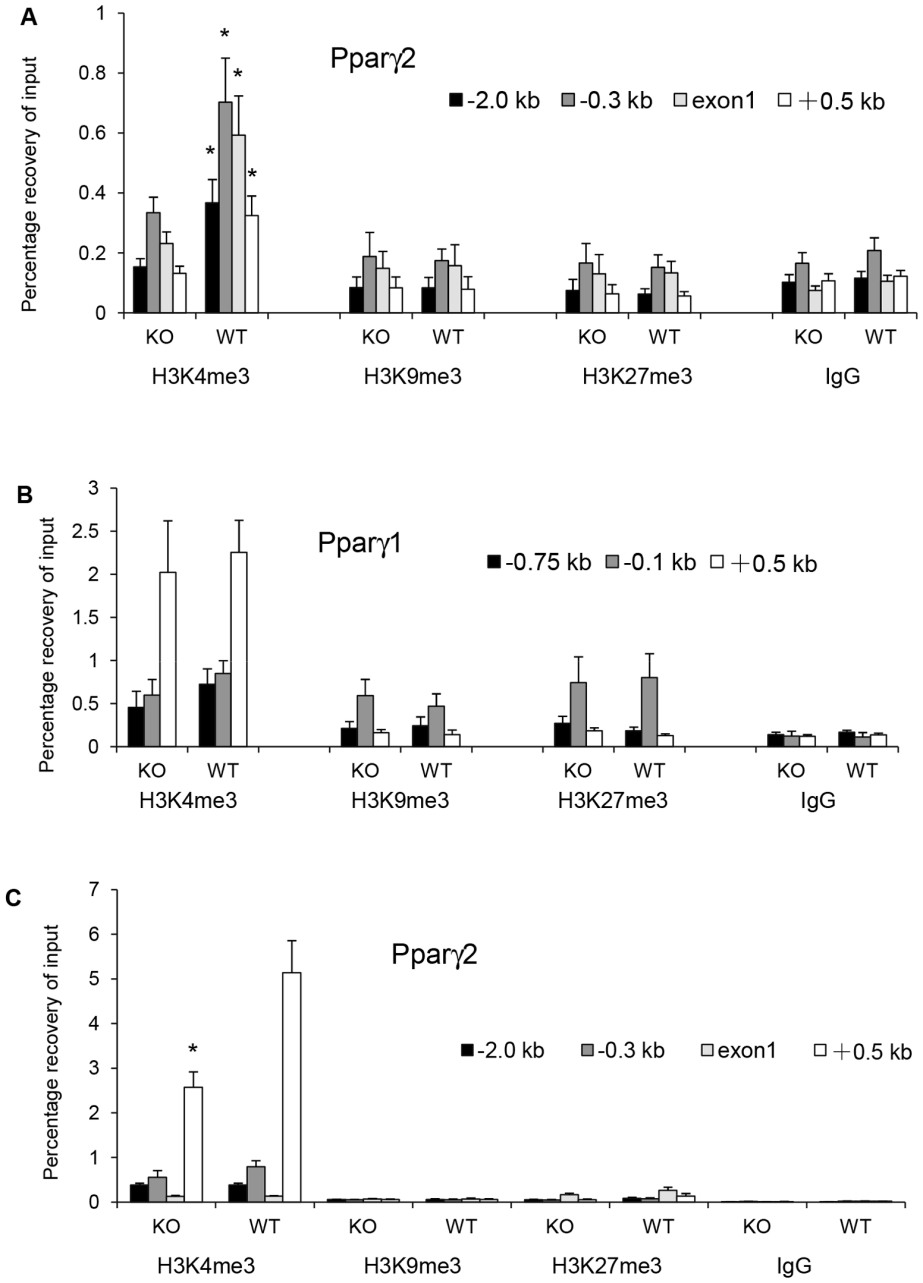
GCN2 programming of PPARγ2 during perinatal development is associated with histone 3 trimethylation. (A). ChIP assay of H3K4me3, H3K9me3 and H3K27me3 on *Pparγ2* promoters in livers of 3 weeks old *Gcn2 KO* (KO) and wild type (WT) mice. −2.0 kb, −0.3 kb, exon1, +0.5 kb indicated locations of primers used for PCR quantification of ChIP assays in the *Pparγ2* promoter region. (Mean ± SEM, n = 4, *p<0.05 *Gcn2 KO vs.* WT). (B). ChIP assay of H3K4me3, H3K9me3 and H3K27me3 on *Pparγ1* promoters in livers of 3 weeks old *Gcn2 KO* (KO) and wild type (WT) mice. −0.75 kb, −0.1 kb, +0.5 kb indicated locations of primers used for PCR quantification of ChIP assays in the *Pparγ1* promoter region. (Mean ± SEM, n = 4). (C). ChIP assay of H3K4me3, H3K9me3 and H3K27me3 on *Pparγ2* promoters in livers of 8 months old Gcn2 KO (KO) and wild type (WT) mice. −2.0 kb, −0.3 kb, exon1, +0.5 kb indicated locations of primers used for PCR quantitation of ChIP assays in *Pparγ2* promoter regions. (mean ± SEM, n = 4, *p<0.05 *Gcn2 KO* vs. WT).

To determine if epigenetic modification of the *Pparγ* gene may underlie the GCN2-dependent programming of the liver, we measured the level of tri-methylation of lysine residues 4, 9, and 27 of histone 3 (H3K4me3, H3K9me3, and H3K27me3) surrounding the chromatin of the *Pparγ2* and *Pparγ1* promoters in three-week-old mice. Weanling mice were chosen because although the perinatal programming is likely to be fixed at this age, the suppression of *Pparγ2, Fsp27*, and *Cidea* mRNA expression in *Gcn2 KO* mice is not observed yet. H3K4me3 is suppressed at all four locations surrounding the *Pparγ2* promoter in the liver of *Gcn2 KO* mice compared to wildtype mice (Fig. 7A). No differences were observed for H3K9 or H3K27 trimethylation in regions surrounding the *Pparγ2* promoter. In contrast, trimethylation of H3K4 of the chromatin surrounding the *Pparγ1* promoter showed no differences between *Gcn2* genotypes (Fig. 7B). When mice reach the age of 8 months, H3K4me3 surrounding the *Pparγ2* promoter increased in both genotypes, but the genotypic difference in trimethylation persisted in the region 500 bp downstream of the *Pparγ2* promoter (Fig. 7C).

### GCN2-dependent perinatal programming of hepatic fat storage is controlled by the brain

**Figure 8:**
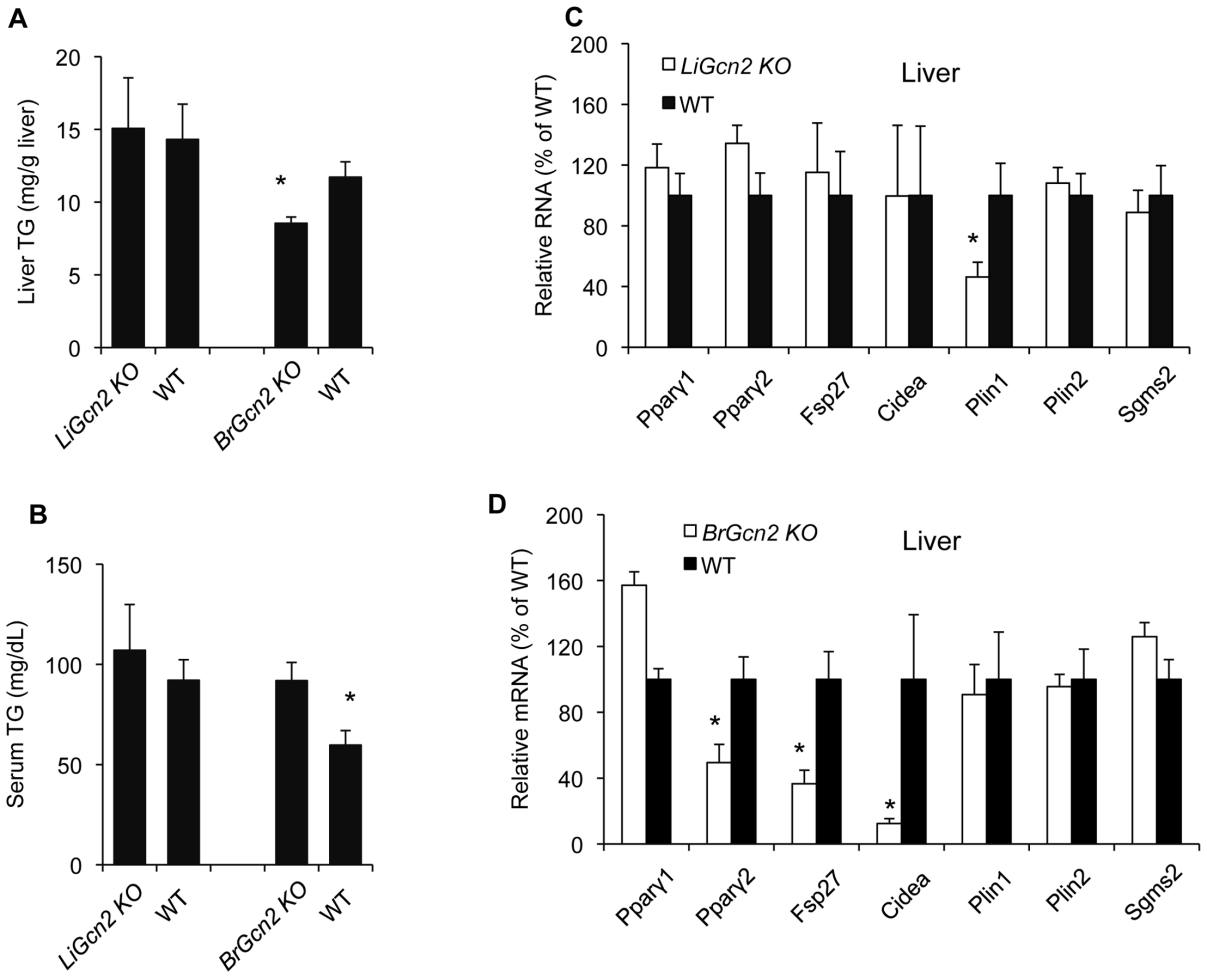
GCN2 expressed in the brain is responsible for PPARγ2, FSP27, and CIDEA and triglyceride storage programming in the liver. (A). Liver triglyceride of *liver-specific Gcn2* knockout mice (*LiGcn2 KO*), *brain-specific Gcn2* knockout (*BrGcn2 KO*) and wildtype (WT) mice (mean ± SEM, n = 8, *p<0.05, *BrGcn2 KO* vs. WT). Total expression of wildtype Gcn2 mRNA was reduced by approximately 65% in the liver of the *LiGcn2 KO* mice and 74.7% in the brain of the *BrGcn2 KO* mice. Residual expression of Gcn2 mRNA in tissues that Gcn2 has been specifically deleted is likely to be due to expression of Gcn2 mRNA in minor cell type for which the Cre-driver transgene is not expressed. No reduction of wild-type Gcn2 mRNA was seen in the liver of the *BrGcn2KO* mice. (B). Serum triglyceride of mice of indicated genotypes (mean ± SEM, n = 8, *p<0.05, *BrGcn2 KO* vs. WT). (C). Expression of *Pparγ1*, *Pparγ2, Fsp27*, *Cidea*, *Plin1, Plin2 and Sgms2* mRNAs in livers of liver-specific knockout (*LiGcn2* KO) and wildtype (WT) mice (normalized to WT mice, mean ± SEM, n = 8, *p<0.05, *LiGcn2 KO* vs. WT).(D). Expression of *Pparγ1*, *Pparγ2, Fsp27*, *Cidea*, *Plin1, Plin2 and Sgms2* mRNAs in livers of brain-specific knockout mice (*BrGcn2 KO*) and wildtype (WT) mice (normalized to WT mice, mean ± SEM, n = 8, *p<0.05, *BrGcn2 KO* vs. WT).

**Figure 9:**
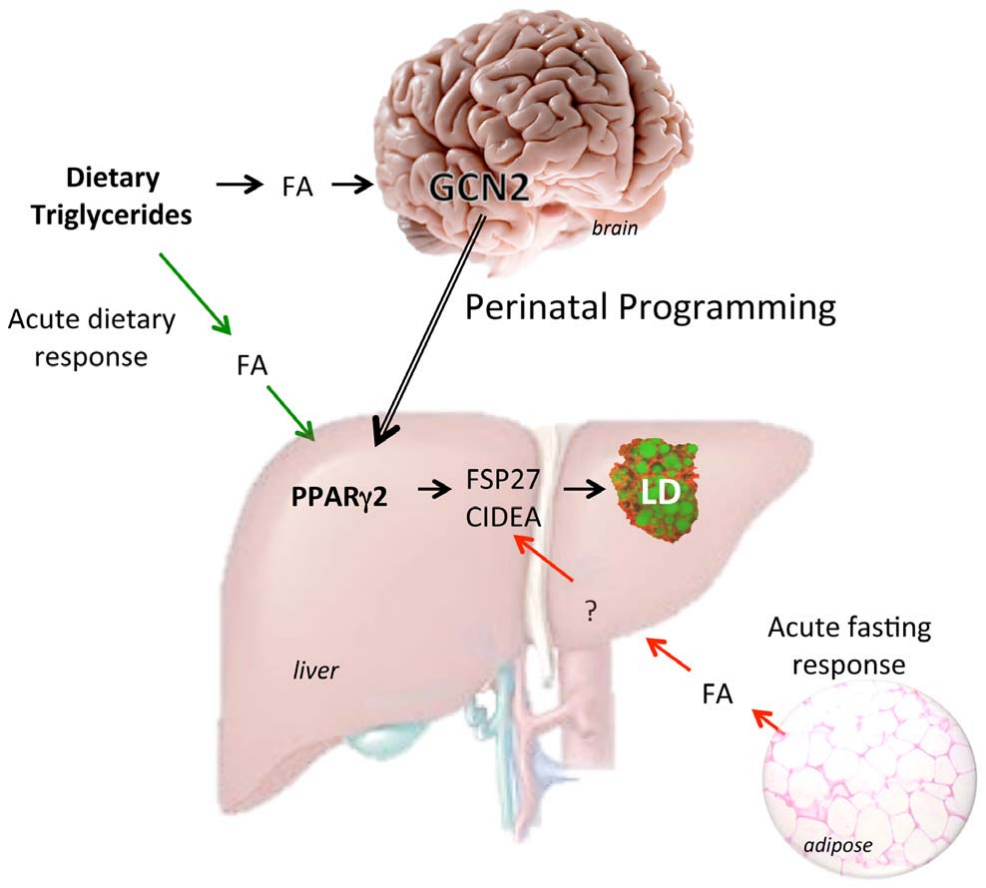
Model for regulation and programming of hepatic triglyceride storage. Dietary triglycerides increase free fatty acids (FA) in circulation that are taken up by the liver and acutely activate the *Pparγ2* gene that in turn induces the expression of the lipid droplet genes *Fsp27* and *Cidea* and increases triglyceride storage. During perinatal development GCN2 in the developing brain senses lipid concentrations provided maternally and signals the liver to program *Pparγ2* for future triglyceride storage levels. GCN2 programming of *Pparγ2* will specifically dictate the future expression of FSP27 and CIDEA and hepatic triglyceride storage but not that of adipose tissue.

The HiS/LoH TG phenotype of *Gcn2* deficient mice stems from dysfunctions in regulating hepatic triglyceride storage, and therefore we speculated that ablating expression of *Gcn2* specifically in the liver would recapitulate this phenotype. Unexpectedly, *liver-specific Gcn2 KO* (*LiGcn2 KO*) reared on the MFC diet failed to display the HiS/LoH TG phenotype, nor was the expression of *Pparγ2, Fsp27,* and *Cidea* altered (Fig. 8A–C). Because *Gcn2 KO* mice displays some behavioral and nutritional sensing defects associated with the brain, we decided to also examine *brain-specific Gcn2 KO* (*BrGcn2 KO*) mice. *BrGcn2 KO* mice reared on the MFC diet displayed the HiS/LoH TG phenotype (Fig. 8A–B), and *Pparγ2, Fsp27*, and *Cidea* were substantially reduced in the liver (Fig. 8D) to a similar degree as seen in global *Gcn2 KO* mice. *Pparγ2, Fsp27*, and *Cidea* expression was not altered in adipose tissue or brain in *BrGcn2 KO* (Fig. S3C–D), consistent with the hypothesis that failure to induce these genes in the liver, and not elsewhere, is the cause of the failure to enhance hepatic triglyceride storage. To confirm that *Gcn2* gene expression was ablated in the brain and not the liver, we examined the expression of *Gcn2* mRNA by qRTPCR using primers specific for exon-12, which should be deleted through the action of the Cre recombinase on the floxed allele of *Gcn2* [37]. Exon-12 *Gcn2* mRNA was reduced by 74.3% in the brain (P<0.001) whereas no reduction was seen in the liver. We conclude that GCN2, or a factor regulated by GCN2, acts as a lipid sensor in the brain and then programs the *Pparγ2* gene in the liver to regulate the lipid droplet protein FSP27 and CIDEA and triglyceride storage (Fig. 9).

Leptin and leptin signaling are known to regulate fat metabolism in part through communication between adipose tissue, the brain, and liver [48], [49]. Although we found leptin levels were reduced and food intake was increased in *Gcn2 KO* mice (Fig. 1D,E), serum leptin levels were normal in *BrGcn2 KO* (not shown) suggesting that leptin does not mediate GCN2-dependent programming of hepatic triglyceride storage. Moreover, as noted above, a low level of hepatic triglycerides and suppressed *Pparγ2, Fsp27, and Cidea* were seen in *Gcn2 KO* mice that also harbor a block in leptin signaling.

## Discussion

The liver plays a key role in determining the fate of lipids and serves as a transient storage depot for triglycerides in the form of lipid droplets. Triglyceride storage and retrieval in lipid droplets in the liver share common biochemical pathways with adipose tissue [5], [14], [50], but how these pathways are regulated is likely to differ substantially because of the different role that the liver plays in lipid storage. For example, during fasting triglycerides are released from adipose tissue as fatty acids, which are used by peripheral tissues for energy or re-esterified and stored in the liver. Consequently triglyceride levels in adipose tissue decrease whereas liver triglyceride concentrations increase during fasting. On the other hand, excessive consumption of high-energy foods can cause systemic accumulation of triglycerides and fatty acids, resulting in hypertriglyceridemia, non-alcoholic fatty liver disease (NAFLD), and obesity. Failure to properly sequester fat into lipid droplets or enhanced lipolysis of triglycerides can result in an elevation of circulating free fatty acids that in turn promote insulin resistance in peripheral tissues and diabetes [14], [51]. PPARγ is a key regulatory factor that controls lipid metabolism and storage both in the liver and adipose tissue. Among several reported activities, PPARγ positively regulates triglyceride storage in lipid droplets by activating the expression of LD proteins including FSP27 and CIDEA, which dimerize and act to promote lipid droplet formation [6], [11],[14],[52]. PPARγ agonists have been used effectively to treat type 2 diabetes [53] which act in part to promote proper storage of fat into lipid droplets. Liver-specific deficiency of PPARγ, in the leptin mutant obese mouse (*ob/ob*), leads to a unique imbalance in triglycerides characterized by low levels of triglycerides in the liver and serum hypertriglyceridemia [2]. Subsequent studies have demonstrated that this unusual phenotype is caused by reduced levels of FSP27 and CIDEA, which result in reduced triglyceride storage in the liver and shunting excess triglycerides to circulation. Although liver steatosis was reduced in these mice compared to *ob/ob* control mice, hyperglycemia and insulin resistance were greatly exacerbated likely due to increased free fatty acid levels. We found that *Gcn2* deficient mice display the same HiS/LoH triglyceride phenotype and also exhibited increased hyperglycemia and insulin resistance when genetically combined with leptin receptor mutant (*db/db*). *Pparγ2*, *Fsp27*, and *Cidea* were substantially reduced in *Gcn2 KO* and *Gcn2;db DKO* mice. FSP27 protein levels were almost entirely obliterated in *Gcn2;db DKO* mice in contrast to *db/db* single mutant mice, which showed enhanced expression. Examination of other aspects of fat metabolism including lipogenesis, fatty acid oxidation, and lipolysis failed to find differences that could explain the HiS/LoH TG phenotype. Moreover, reduced expression of *Pparγ2*, *Fsp27*, and *Cidea* in *Gcn2 KO* mice was present in the liver but not in adipose tissue where these genes are known to be important for LD metabolism. We conclude that the HiS/LoH TG phenotype in *Gcn2* deficient mice is caused by a specific deficiency in LD formation in the liver that secondarily results in the accumulation of triglycerides in circulation.

Basal hepatic triglyceride storage in *Gcn2* deficient mice is normal when mice are reared on a relatively low fat chow diet. Under these conditions, the expression of *Fsp27* and *Cid*ea is very low in the liver of both wildtype and *Gcn2 KO* mice whereas these genes are expressed at high levels in WAT and/or BAT. Lipid overload or fasting can increase triglyceride levels in the liver, and we showed that this is correlated with the induction of *Fsp27* and *Cidea*. PPARγ is required for the induction of FSP27 and CIDEA when dietary fat is increased or as a consequence of a block in leptin signaling but is not required for their induction during fasting when PPARγ is either repressed or not induced. CREB-H [54] is required for the normal fasting response of FSP27 and CIDEA. We discovered that GCN2 is not required for the fasting response at the adult stage. Intriguingly however, GCN2 is required to program a set point for *Pparγ2*, *Fsp27*, and *Cidea* gene expression when perinatal dietary fat exceeds 13% kcal. We found that exposure of mice to MFC diet only during perinatal development (conception to weaning) was sufficient to program hepatic LD gene expression and triglyceride storage to the high TG storage set-point in adult mice even though these mice consumed LFC for more than 7 months after weaning. However, we showed that this set point could be temporarily over ridden as a consequence of fasting but quickly recovers upon refeeding. Hyperactivation of PPARγ by treatment of *Gcn2 KO* mice with the PPARγ agonist rosiglitazone partially restores normal FSP27, CIDEA, and triglyceride homeostasis supporting the hypothesis that PPARγ is the key factor that mediates GCN2-dependent control of the hepatic triglycerides set point.

Programming the triglyceride storage set point in the liver during early development is likely to be determined by epigenetic modification and numerous reports have linked nutritional programming of metabolic states have been linked to histone and/or DNA modifications [30], [55]. Indeed, we found that significant induction of H3K4 trimethylation of the *Pparγ2* promoter region in newly weaned mice occurs six months before elevated expression of *Pparγ2* and its downstream targets *Fsp27* and *Cidea* are detected in wildtype, but not in *Gcn2 KO* mice. H3K4me3 can either mark an active promoter or a promoter that is poised for transcription [56], and we suggest that elevated H3K4me3 surrounding the *Pparγ2* promoter in neonatal mice signifies its potential for transcriptional activation later in life. In contrast, H3K4 trimethylation surrounding the *Pparγ1* promoter showed no genotypic differences consistent with similar *Pparγ1* gene expression levels. The *Pparγ2* promoter was previously noted as nutritionally sensitive in adipose tissue [57]. Our studies extend this conclusion to the liver and show that *Pparγ2* can be programmed by perinatal nutrition.

We speculated that the HiS/LoH TG phenotype of the *Gcn2 KO* mice, which primarily arises from misregulated hepatic triglyceride storage, was caused by the lack of GCN2 activity in the liver. Surprisingly, liver-specific ablation of *Gcn2* in the liver failed to recapitulate the HiS/LoH TG phenotype whereas ablation of *Gcn2* in the brain resulted in a HiS/LoH TG phenotype including a potent reduction in *Pparγ2*, *Fsp27*, and *Cidea*. Thus GCN2 in the perinatal brain is remotely programming the expression of *Pparγ2*, *Fsp27*, and *Cidea* in the liver. This programming appears to be specific for the liver as no differences in expression of these genes were seen in the hypothalamus or WAT. GCN2 is already known to have two other functions in the brain including sensing deficiencies in dietary essential amino acids in the piriform cortex and regulating aversive feeding behavior [38], [39], and GCN2 modulates hippocampus-dependent long-term memory [58]. The hypothalamus is the major center in the brain that modulates metabolic functions in peripheral organs including the liver, and therefore it is the most likely site of GCN2-dependent programming of hepatic triglyceride storage. GCN2 is known to be activated by uncharged tRNAs associated with amino acid deprivation [59], specific viral RNAs [60], or UV irradiation [61] but how it functions in this new role as a lipid sensor is unknown. We speculate that either GCN2 is directly activated by a specific fatty acid associated with the MFC diet or that increased dietary fat causes a localized amino acid deprivation in the brain, which then results in increased concentration of uncharged tRNAs and activation of GCN2.

Aspects of glucose and fat metabolism are coordinated by both afferent and efferent signaling through the hepatic branch of the vagus nerve [62], [63]. The expression of PPARγand FSP27 in the liver has also been shown to modulate blood pressure via afferent vagal signals from the liver [64]. We speculate that hepatic PPARγ is a key regulator of both efferent signals sent from the brain to the liver, as in the case of perinatal programming, and afferent signals that inform the central nervous system of the metabolic state of the liver.

Although a low level of fat storage in the liver is considered to be normal, our discovery that hepatic triglyceride storage is programmed during perinatal development in accord with dietary fat suggests that altering the basal level of fat storage may serve an adaptive function. We speculate that since dietary fat content is highly variable and dependent upon locally available food sources, it may be adaptive to set the future basal hepatic fat storage level in accordance with the diet experienced during the perinatal period. We propose that GCN2 is a key regulatory factor in programming the “thrifty phenotype” [65] which promotes efficient energy storage and utilization. Programming hepatic triglyceride storage also has consequence to glucose homeostasis as we found that a reduced capacity to store triglycerides in the liver is associated with more rapid and severe progression to diabetes in leptin receptor mutant mice.

## Supporting Information

Figure S1 **Lipolysis and lipid uptake and transport are normal in** ***Gcn2 KO*** **mice related to** **Fig. 1** **.** (A). Glycerol release as representative of lipolysis activities from isolated adipocytes of wild type (WT) and *Gcn2 KO* (KO) mice after 24-hour or 72-hour fasting (mean ± SEM, n = 8). (B). Lipolipase activities in livers of mice of indicated genotypes in random fed or 24-hour fasting state (mean ± SEM, n = 8). (C). Lipolipase activities in adipose tissues of mice of indicated genotypes in random fed or 24-hour fasting state (mean ± SEM, n = 8). (D). Lipolipase activities in muscle of mice of indicated genotypes in random fed or 24-hour fasting state (mean ± SEM, n = 8). (E). Expression of fatty acid uptake related genes (*Fabp, Fatp, Cd36, Lpl*) in livers of mice of indicated genotypes in random fed, 24 hr and 72 hr fasting state (normalized to random fed WT mice, mean ± SEM, n = 4). (F). Expression of *Apob, Apoe, Apoa1* and *Apoc1* mRNAs in livers of mice of indicated genotypes in random fed, 24 hr and 72 hr fasting state (normalized to random fed WT mice, mean ± SEM, n = 4).
(TIF)

Figure S2 **Hepatic oxidation and synthesis of fatty acids in the liver are nearly normal in** ***Gcn2 KO*** **mice related to** **Fig. 2** **.** (A). Peroxisomal and total fatty acid β-oxidation activities in livers of fed and fasted wild type (WT) and *Gcn2 KO* (KO) mice (mean ± SEM, n = 8, *p<0.05 *Gcn2 KO vs.* WT). (B). Expression of mRNAs of microsomal and peroxisomal oxidation related genes (*Cyp4a14, Cyp4a10, Cyp2b10, Aldh3a2, Cyp4a12a, Acaa1a, Ehhadh*) in livers of mice of indicated genotypes in random fed state (normalized to WT mice, mean ± SEM, n = 8, *p<0.05 *Gcn2 KO vs.* WT). (C). Expression of mRNAs of mitochondrial oxidation related genes (*Pparα, Cpt1a, Acox1, Acadlv, Acadl, Acadm*) in livers of fed and fasted mice of indicated genotypes (normalized to random fed WT mice, mean ± SEM, n = 8). (D). PPARα protein from liver lysates of mice of indicated genotypes (left, western blot; right, PPARα protein relative to tubulin and normalized to WT fed mice, mean ± SEM, n = 3). (E). Expression of mRNAs of fatty acid synthesis related genes (*Srebp1c, Srebp1a, Fas, Scd, Acc1*) in livers of fed and fasted mice of indicated genotypes (normalized to random fed WT mice, mean ± SEM, n = 8).
(TIF)

Figure S3 **Expression of genes associated with triglyceride storage related to** **Fig. 2** **and** **4** **.** (A). Expression of *Pparγ1*, *Pparγ2, Fsp27*, *Cidea*, *Plin1, Plin2 and Sgms2* mRNAs in adipose tissues of wild type (WT) and *Gcn2 KO* (KO) mice, 8 months of age, (normalized to WT mice, mean ± SEM, n = 8, *p<0.05 *Gcn2 KO vs.* WT). (B). Expression of *Pparγ1*, *Pparγ2, Fsp27*, *Cidea*, *Plin1, Plin2 and Sgms2* mRNAs in adipose tissues of mice of indicated genotypes (normalized to *db/db* mice, mean ± SEM, n = 8, *p<0.05, *Gcn2;db DKO* vs. *db/db*). (C). Expression of *Pparγ1*, *Pparγ2, Fsp27*, *Cidea*, *Plin1, Plin2 and Sgms2* mRNAs in adipose tissues of brain-specific knockout mice (*BrGcn2 KO*) and wildtype (WT) mice (normalized to WT mice, mean ± SEM, n = 4, *p<0.05, *BrGcn2 KO* vs. WT). (D). Expression of *Pparγ1*, *Pparγ2, Fsp27*, *Cidea*, *Plin1, Plin2 and Sgms2* mRNAs in brains of brain-specific knockout mice (*BrGcn2 KO*) and wildtype (WT) mice (normalized to WT mice, mean ± SEM, n = 4).
(TIF)

Table S1 **Phenotypic and metabolic parameters for** ***Gcn2 KO*** **and WT mice in fed or fasting state.** *Gcn2 KO* and WT mice, 6–8 months old, were fed MFC diet or fasted for 24 hrs or 72 hrs. Random fed mice, n≥8 (male) per genotype; for fasting 24 hours, n≥16 (male) per genotype; for fasting 72 hrs, n≥8 (male) per genotype. Values represent mean ± SEM. *p<0.05 and **p<0.001 by two-tailed Student's t test for *Gcn2 KO* versus WT mice in either random fed or fasting state.
(TIF)

Table S2 **Primers used for real time PCR of mRNA levels in this article.** All primer sequences are listed from 5′ to 3′.
(TIF)
