# Supplementary figures and images for: GCN2 in the Brain Programs PPARγ2 and Triglyceride Storage in the Liver during Perinatal Development in Response to Maternal Dietary Fat
Source: PLoS One. 2013 Oct 10;8(10):e75917. doi: 10.1371/journal.pone.0075917 (PMC3794936; doi:10.1371/journal.pone.0075917)

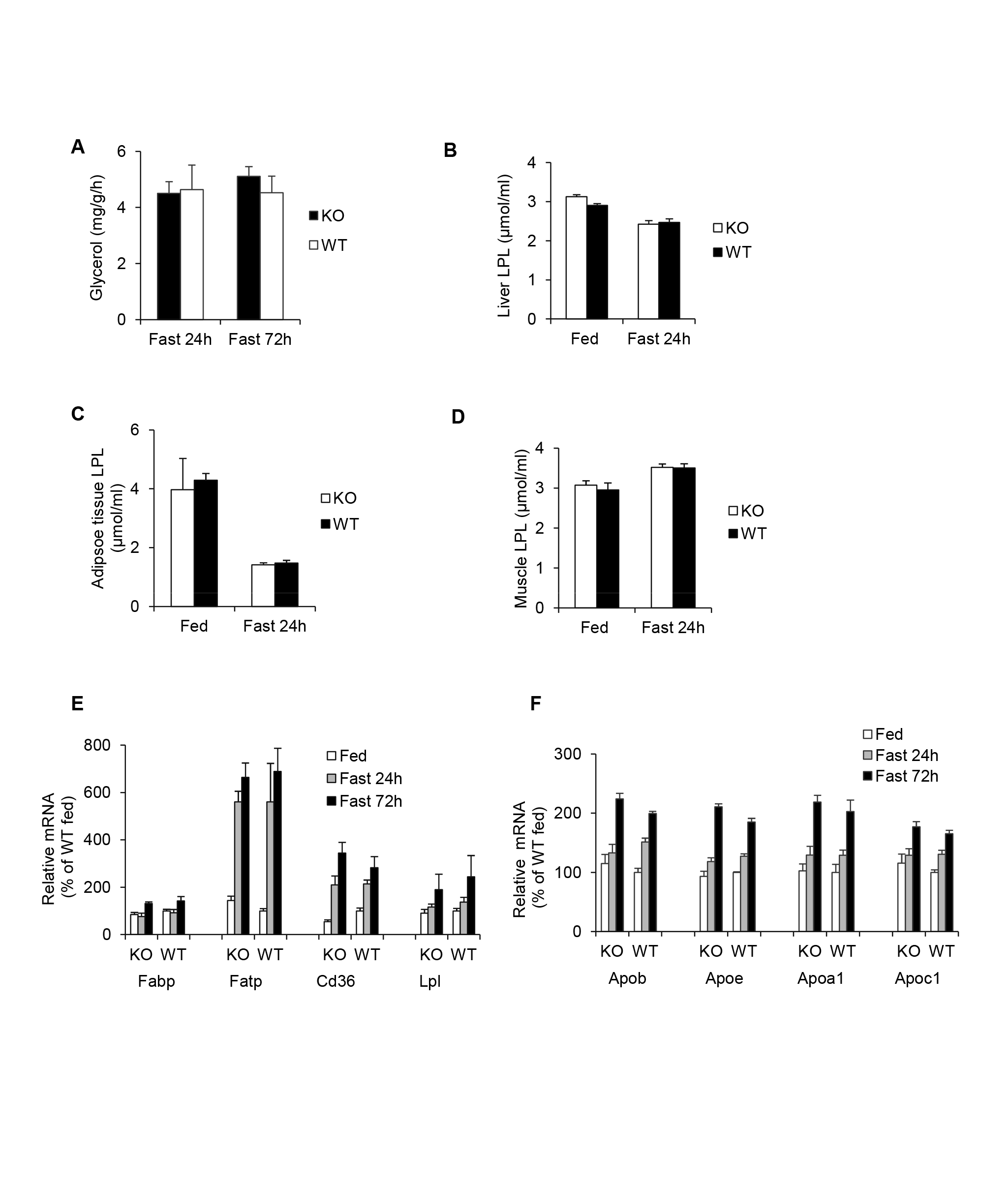

Supplement: Figure S1 — Lipolysis and lipid uptake and transport are normal in Gcn2 KO mice related to Fig. 1 . (A). Glycerol release as representative of lipolysis activities from isolated adipocytes of wild type (WT) and Gcn2 KO (KO) mice after 24-hour or 72-hour fasting (mean ± SEM, n = 8). (B). Lipolipase activities in livers of mice of indicated genotypes in random fed or 24-hour fasting state (mean ± SEM, n = 8). (C). Lipolipase activities in adipose tissues of mice of indicated genotypes in random fed or 24-hour fasting state (mean ± SEM, n = 8). (D). Lipolipase activities in muscle of mice of indicated genotypes in random fed or 24-hour fasting state (mean ± SEM, n = 8). (E). Expression of fatty acid uptake related genes (Fabp, Fatp, Cd36, Lpl) in livers of mice of indicated genotypes in random fed, 24 hr and 72 hr fasting state (normalized to random fed WT mice, mean ± SEM, n = 4). (F). Expression of Apob, Apoe, Apoa1 and Apoc1 mRNAs in livers of mice of indicated genotypes in random fed, 24 hr and 72 hr fasting state (normalized to random fed WT mice, mean ± SEM, n = 4). (TIF) [file pone.0075917.s001.tif]

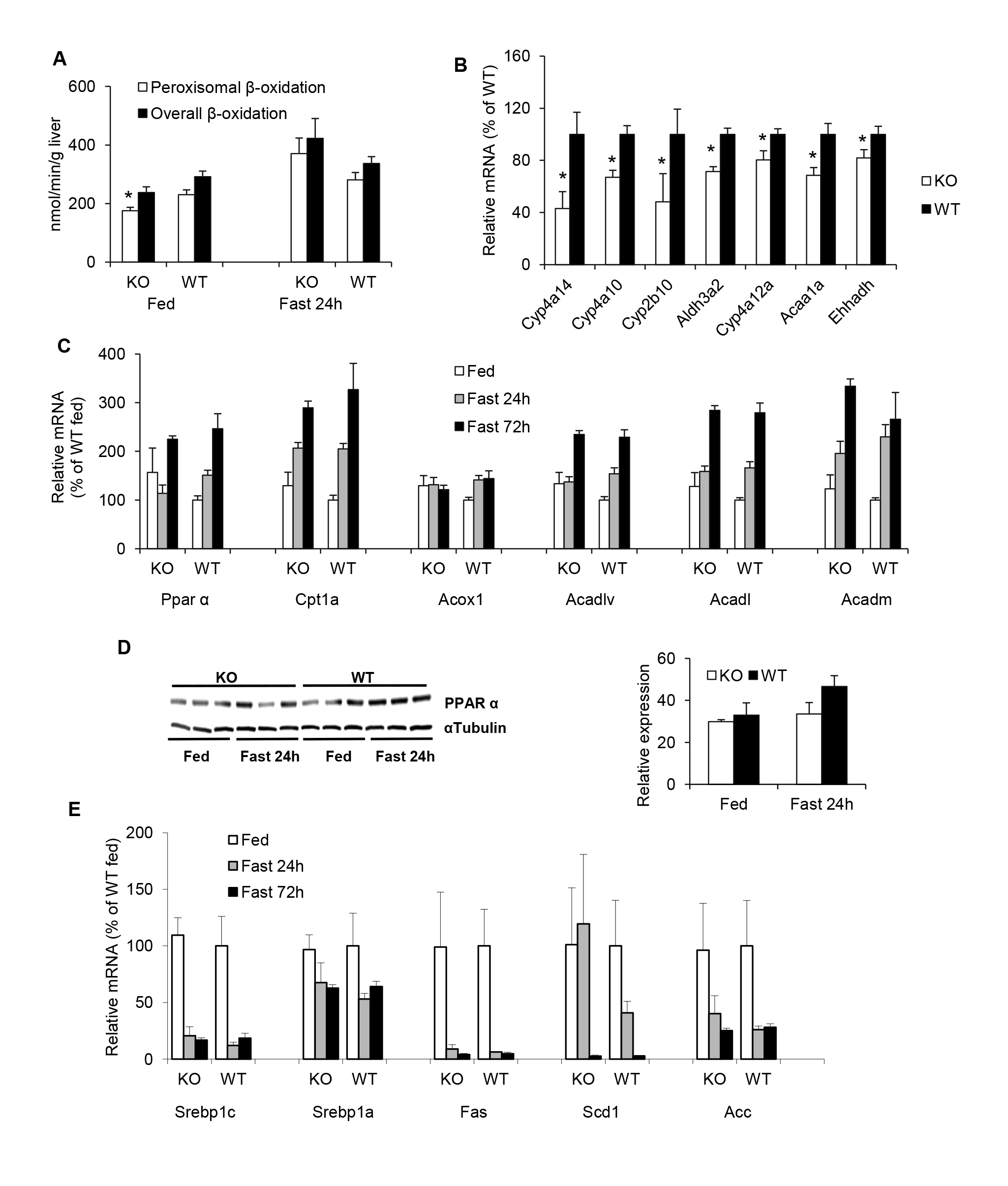

Supplement: Figure S2 — Hepatic oxidation and synthesis of fatty acids in the liver are nearly normal in Gcn2 KO mice related to Fig. 2 . (A). Peroxisomal and total fatty acid β-oxidation activities in livers of fed and fasted wild type (WT) and Gcn2 KO (KO) mice (mean ± SEM, n = 8, *p<0.05 Gcn2 KO vs. WT). (B). Expression of mRNAs of microsomal and peroxisomal oxidation related genes (Cyp4a14, Cyp4a10, Cyp2b10, Aldh3a2, Cyp4a12a, Acaa1a, Ehhadh) in livers of mice of indicated genotypes in random fed state (normalized to WT mice, mean ± SEM, n = 8, *p<0.05 Gcn2 KO vs. WT). (C). Expression of mRNAs of mitochondrial oxidation related genes (Pparα, Cpt1a, Acox1, Acadlv, Acadl, Acadm) in livers of fed and fasted mice of indicated genotypes (normalized to random fed WT mice, mean ± SEM, n = 8). (D). PPARα protein from liver lysates of mice of indicated genotypes (left, western blot; right, PPARα protein relative to tubulin and normalized to WT fed mice, mean ± SEM, n = 3). (E). Expression of mRNAs of fatty acid synthesis related genes (Srebp1c, Srebp1a, Fas, Scd, Acc1) in livers of fed and fasted mice of indicated genotypes (normalized to random fed WT mice, mean ± SEM, n = 8). (TIF) [file pone.0075917.s002.tif]

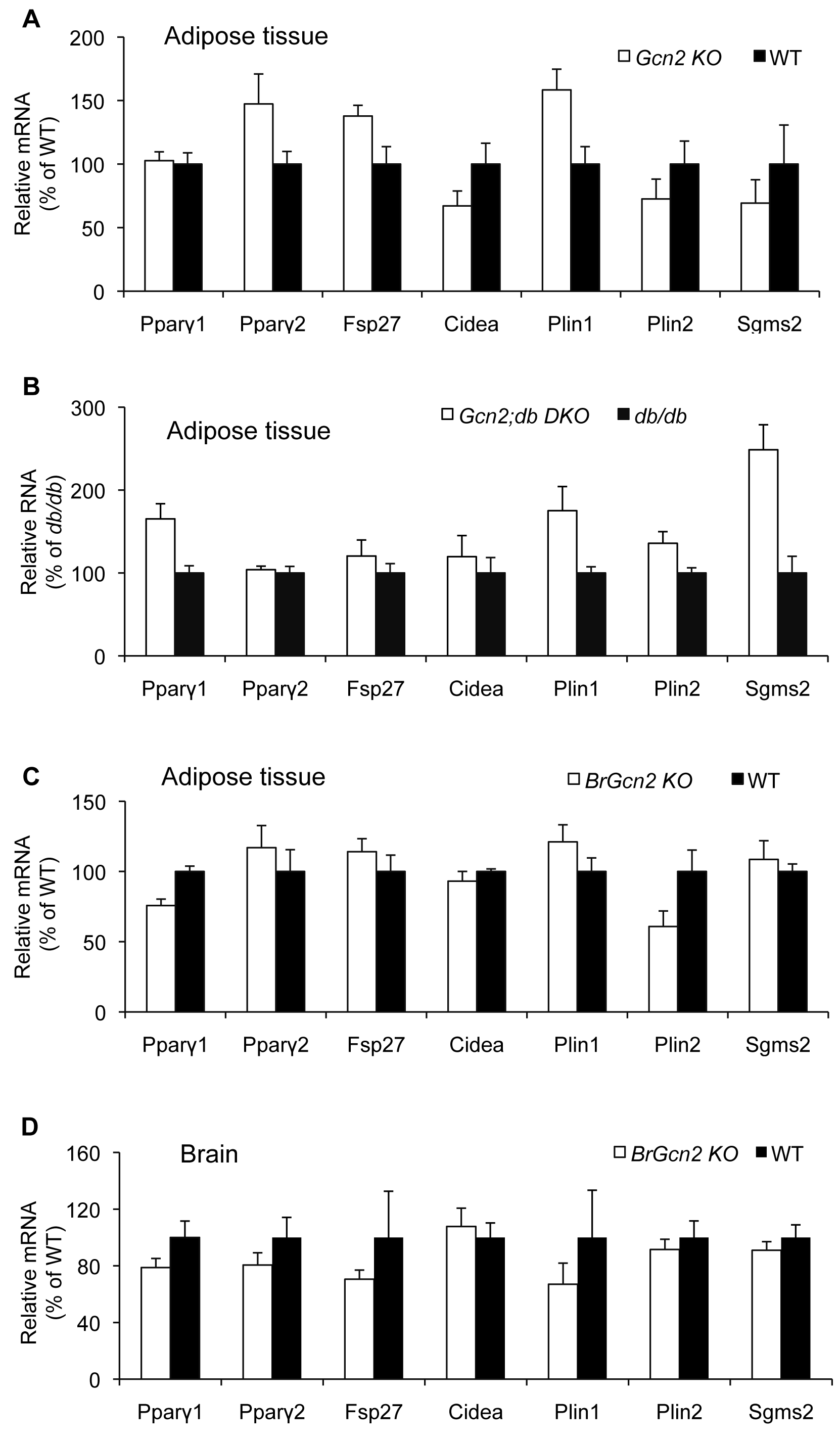

Supplement: Figure S3 — Expression of genes associated with triglyceride storage related to Fig. 2 and 4 . (A). Expression of Pparγ1, Pparγ2, Fsp27, Cidea, Plin1, Plin2 and Sgms2 mRNAs in adipose tissues of wild type (WT) and Gcn2 KO (KO) mice, 8 months of age, (normalized to WT mice, mean ± SEM, n = 8, *p<0.05 Gcn2 KO vs. WT). (B). Expression of Pparγ1, Pparγ2, Fsp27, Cidea, Plin1, Plin2 and Sgms2 mRNAs in adipose tissues of mice of indicated genotypes (normalized to db/db mice, mean ± SEM, n = 8, *p<0.05, Gcn2;db DKO vs. db/db). (C). Expression of Pparγ1, Pparγ2, Fsp27, Cidea, Plin1, Plin2 and Sgms2 mRNAs in adipose tissues of brain-specific knockout mice (BrGcn2 KO) and wildtype (WT) mice (normalized to WT mice, mean ± SEM, n = 4, *p<0.05, BrGcn2 KO vs. WT). (D). Expression of Pparγ1, Pparγ2, Fsp27, Cidea, Plin1, Plin2 and Sgms2 mRNAs in brains of brain-specific knockout mice (BrGcn2 KO) and wildtype (WT) mice (normalized to WT mice, mean ± SEM, n = 4). (TIF) [file pone.0075917.s003.tif]

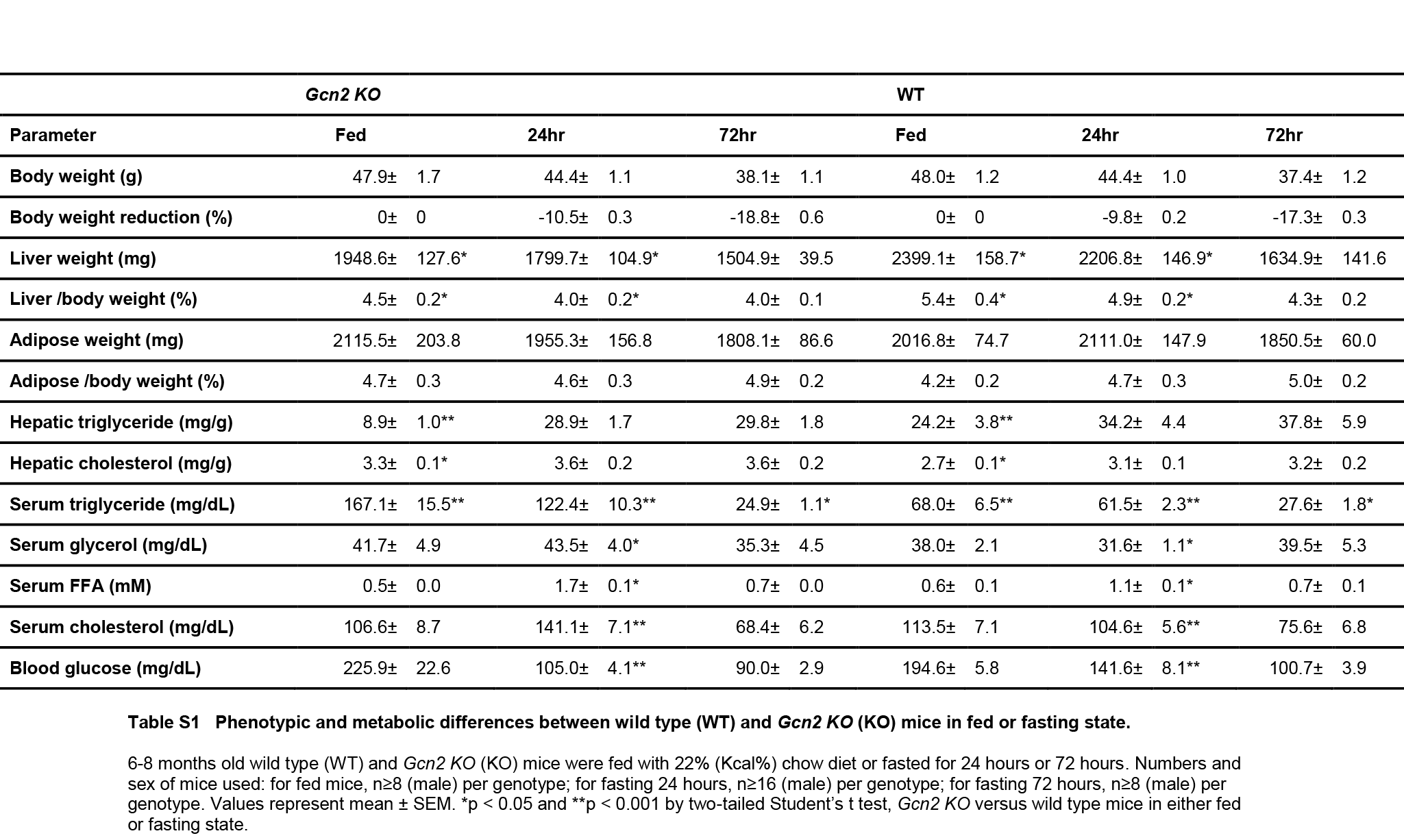

Supplement: Table S1 — Phenotypic and metabolic parameters for Gcn2 KO and WT mice in fed or fasting state. Gcn2 KO and WT mice, 6–8 months old, were fed MFC diet or fasted for 24 hrs or 72 hrs. Random fed mice, n≥8 (male) per genotype; for fasting 24 hours, n≥16 (male) per genotype; for fasting 72 hrs, n≥8 (male) per genotype. Values represent mean ± SEM. *p<0.05 and **p<0.001 by two-tailed Student's t test for Gcn2 KO versus WT mice in either random fed or fasting state. (TIF) [file pone.0075917.s004.tif]

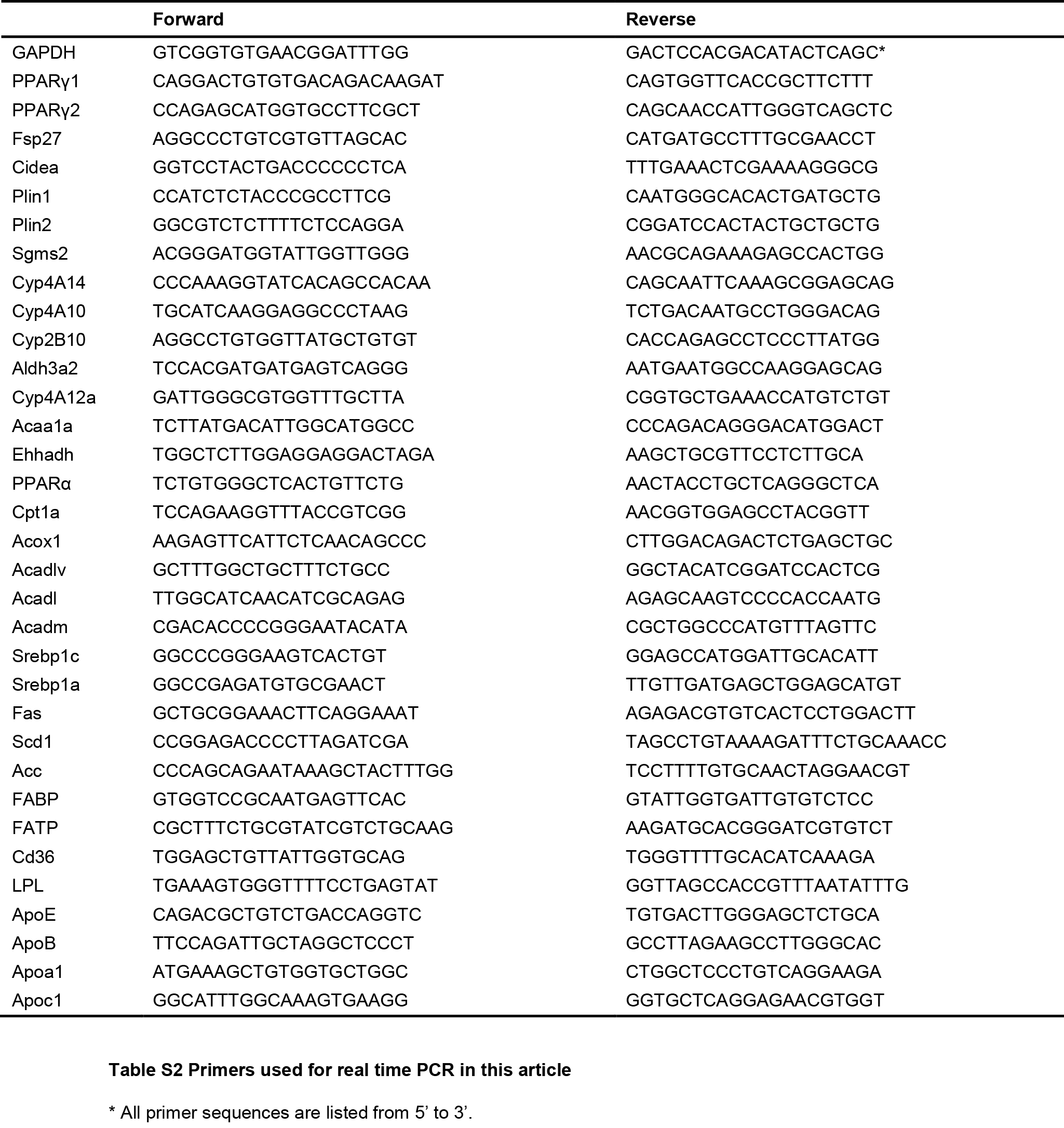

Supplement: Table S2 — Primers used for real time PCR of mRNA levels in this article. All primer sequences are listed from 5′ to 3′. (TIF) [file pone.0075917.s005.tif]
